# Mounting, structure and autocleavage of a type VI secretion-associated Rhs polymorphic toxin

**DOI:** 10.1038/s41467-021-27388-0

**Published:** 2021-12-01

**Authors:** Dukas Jurėnas, Leonardo Talachia Rosa, Martial Rey, Julia Chamot-Rooke, Rémi Fronzes, Eric Cascales

**Affiliations:** 1 Laboratoire d’Ingénierie des Systèmes Macromoléculaires (LISM), Institut de Microbiologie, Bioénergies et Biotechnologie (IM2B), Aix-Marseille Université – CNRS, UMR 7255, Marseille, France; 2 Structure and Function of Bacterial Nanomachines, Institut Européen de Chimie et Biologie, Univ. Bordeaux – CNRS, UMR 5234 Microbiologie Fondamentale et Pathogénicité, 33600 Pessac, France; 3 Mass Spectrometry for Biology Unit, Department of Structural Biology and Chemistry, Institut Pasteur – CNRS, USR 2000, 75015 Paris, France

**Keywords:** Biochemistry, Bacterial toxins, Cryoelectron microscopy

## Abstract

Bacteria have evolved toxins to outcompete other bacteria or to hijack host cell pathways. One broad family of bacterial polymorphic toxins gathers multidomain proteins with a modular organization, comprising a C-terminal toxin domain fused to a N-terminal domain that adapts to the delivery apparatus. Polymorphic toxins include bacteriocins, contact-dependent growth inhibition systems, and specialized Hcp, VgrG, PAAR or Rhs Type VI secretion (T6SS) components. We recently described and characterized Tre23, a toxin domain fused to a T6SS-associated Rhs protein in *Photorhabdus laumondii*, Rhs1. Here, we show that Rhs1 forms a complex with the T6SS spike protein VgrG and the EagR chaperone. Using truncation derivatives and cross-linking mass spectrometry, we demonstrate that VgrG-EagR-Rhs1 complex formation requires the VgrG C-terminal β-helix and the Rhs1 N-terminal region. We then report the cryo-electron-microscopy structure of the Rhs1-EagR complex, demonstrating that the Rhs1 central region forms a β-barrel cage-like structure that encapsulates the C-terminal toxin domain, and provide evidence for processing of the Rhs1 protein through aspartyl autoproteolysis. We propose a model for Rhs1 loading on the T6SS, transport and delivery into the target cell.

## Abstract (web summary)

Rearrangement hot spots (Rhs) proteins are bacterial polymorphic toxin systems. Here, the authors show that Rhs1 forms a complex with the Type VI secretion system (T6SS) spike protein VgrG and the EagR chaperone. They also present the cryo-EM structure of the Rhs1-EagR complex and propose a model for Rhs loading and delivery by the T6SS.

## Introduction

To colonize a niche or a host, bacteria deliver toxins that destroy bacterial rivals or hijack the host defenses. Polymorphic toxins are defined as multidomain proteins with a modular organization, composed of a C-terminal toxin domain fused to a N-terminal trafficking domain^1,2^. Polymorphic toxins comprise diverse families including bacteriocins, contact-dependent growth inhibition (CDI) CdiA, Multiple adhesin family (Maf), Rearrangement hot spots (Rhs), and specialized type VI secretion system (T6SS) Hcp, VgrG, and PAAR proteins^1–9^.

Rhs proteins have a tripartite architecture: a N-terminal region (Rhs^NT^) that specifies the mode of secretion, a conserved central domain predicted to fold as a shell-like structure (Rhs^Shell^) consisting of Tyrosine/Aspartate-rich (YD) YDxxxGRL(I/T) repeats demarcated from a highly variable C-terminal toxin domain (Rhs^CT^) by a PxxxxDPxGL motif^1,10^. Rhs^CT^ domains possess various activities including nucleases, metallopeptidases, ADP-ribosylases, or deaminases^1,2^. These Rhs^CT^ domains share GC content and codon bias that are different from sequences encoding Rhs^Shell^, suggesting frequent rearrangements by recombination^11–13^. Genes encoding Rhs polymorphic toxins are usually genetically linked to a gene encoding a specific immunity protein required to confer self-protection against the cognate Rhs^CT ^^1,2,8^, suggesting an antibacterial activity of the Rhs^CT^ toxin domain. Indeed, Rhs proteins have been shown to confer advantages against bacterial competitors to *Dickeya dadantii*, *Proteus mirabilis*, *Pseudomonas aeruginosa*, *Serratia marcescens*, *Enterobacter cloacae*, *Aeromonas dhakensis*, *P. fluorescens*, and several pathogenic *E. coli* strains^6,10,14–22^.

YD-repeat-like elements are not only found in proteobacteria but are widely distributed in Bacteria, Archaea, and Eukarya, suggesting that they correspond to an ancient fold that has been adapted to various needs^23–26^. Well-known examples of YD-repeat proteins are the insecticidal TcB/TcC complex found in entomopathogenic strains such as *Photorhabdus*, *Yersinia*, and *Serratia* that associate with a third subunit, TcA, to assemble a tripartite Tc-toxin complex targeting insect cells^27,28^, the antibacterial WapA tRNases encoded on the genomes of Gram-positive bacteria, such as *Bacillus* and *Listeria* species^15^, Ss-Rhs1 that is essential for the virulence of the fungus *Sclerotinia sclerotiorum* toward host plants^29^, or the teneurins found in higher metazoans and involved in the establishment of neuronal cell connections during embryogenesis^30–33^. YD-repeat proteins structurally characterized so far, Tc-toxins and teneurins, assemble a large β-barrel shell^31,32,34–36^. While the teneurin C-terminal domain is positioned at the external surface of the shell, the toxin domains of Tc-toxins are encaged into the β-barrel^31,34,35,37^. It is not well determined how the toxin domain is released from the barrel but Rhs and TcC proteins share conserved motifs with the signature of aspartyl proteases that catalyze internal autocleavages^10,38,39^.

The N-terminal regions of Rhs proteins, Rhs^NT^, define their mode of secretion. Rhs proteins can be associated with diverse secretion mechanisms such as type VI (T6SS) or type VII (T7SS) secretion systems^1,2^. However, most *rhs* genes are located within T6SS gene clusters or *hcp-vgrG* islands^1^. Indeed, those Rhs proteins are dependent on the T6SS for their delivery into target cells^6,14–22^. The T6SS is a multiprotein machine that uses a contractile mechanism to inject an effector-loaded needle into target cells^40^. It comprises a membrane complex that serves as a channel for the passage of the needle, on which is docked the assembly platform that initiates the polymerization of the contractile tail and triggers its contraction^41–44^. Effectors are usually fused or associated to components of the needle, such as the Hcp tube protein, the VgrG spike, or PAAR, a small conical subunit that sharpens the tip of the VgrG spike^3,9,45–49^. Rhs^NT^ domains of T6SS-associated Rhs proteins share a PAAR motif (Rhs^PAAR ^^47^), suggesting that Rhs proteins associate with the needle tip through VgrG-Rhs^PAAR^ interactions. Indeed, Rhs^NT^ deletion abolishes Rhs T6SS-dependent secretion in *A. dhakensis*^10^ and the *P. fluorescens*, *S. marcescens*, and *A. dhakensis* Rhs N-terminal domains have been shown to interact with VgrG^10,20,21^. T6SS-associated Rhs^NT^ domains may also bear transmembrane helices that are proposed to insert into the membrane of the target cell and that require a dedicated chaperone of the effector-associated gene (Eag, DUF1795) family to be maintained in a preinsertion state^19–21,50^.

Recently, we characterized a new toxin domain associated to a Rhs element in *Photorhabdus laumondii*^51^. This Rhs1 toxin, encoded by the *plu0353* gene, is delivered by the T6SS, through interactions with VgrG (Plu0355) and the Eag chaperone (EagR, Plu0354). Production of the Plu0353 Rhs1^CT^, called Tre23, is toxic in the cytoplasm of *E. coli* and is counteracted by the Tri23 (Plu0352) immunity protein. Tre23 inhibits protein synthesis through ADP-ribosylation of helix 44 of the ribosomal 23 S RNA, hence preventing protein elongation by blocking the GTPase-associated center. Here we conduct a biochemical and structural characterization of Rhs1. Using a combination of pull-down and cross-link mass spectrometry analyses, we first provide details on the association of the VgrG, Rhs1, and EagR proteins, demonstrating that Rhs1^NT^ and VgrG gp5-like needle are responsible for VgrG-Rhs1 complex formation, and that EagR binds to two regions located upstream and downstream the PAAR motif of Rhs1^NT^, including a predicted hydrophobic transmembrane domain. We then provide the cryo-electron microscopy structure of the Rhs1-EagR complex, demonstrating that Rhs1^Shell^ assembles a β-barrel cage that encapsulates Rhs1^CT^. We finally demonstrate that Rhs1 undergoes autocleavage at two positions located on each side of the Rhs1^Shell^ barrel, releasing the toxin Rhs1^CT^ domain.

## Results

### Organization of the *P. laumondii* VgrG-Rhs1-EagR complex

**Fig. 1:**
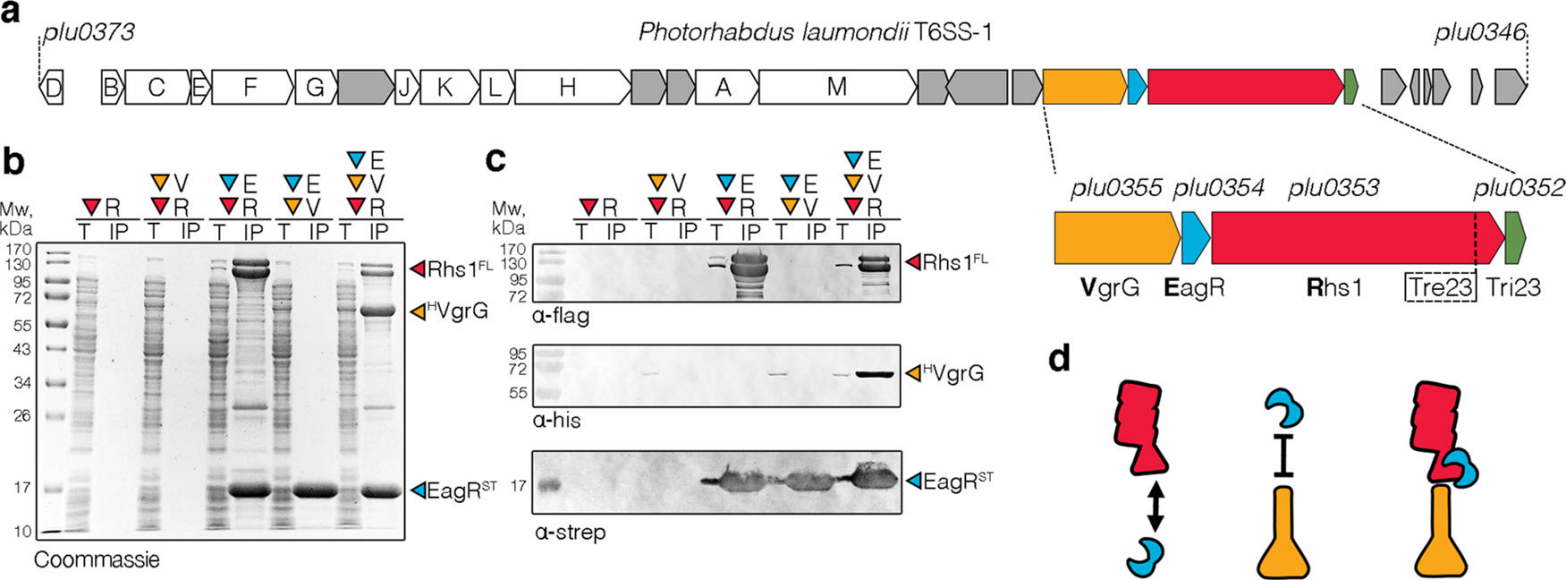
Rhs1 mounting on the VgrG spike. **a** Schematic representation of the *Photorhabdus laumondii* T6SS gene cluster. Genes encoding T6SS core components of T6SS are identified by their Tss corresponding letter (e.g., A for TssA). A close-up emphasizing the *vgrG-eagR-rhs1-imm1* genetic locus is shown below (*vgrG*, orange; *eagR*, blue; *rhs1*, red; *tri23*, green). **b** and **c** Pull-down assays. Total cell extracts (T) from *E. coli* BL21(DE3) cells producing various combinations of His_6_-tagged VgrG (^H^VgrG, V), Strep-tagged EagR (^ST^EagR, E), and FLAG-tagged Rhs1 (Rhs1^FL^, R) were subjected to purification on streptactin-agarose beads. Strep-tagged and co-precipitated proteins were eluted with desthiobiotin (IP). Total and IP fractions were analyzed by SDS-PAGE and co-purified proteins were stained by Coomassie blue (**b**) or immunodetected using anti-His, anti-Strep, and anti-FLAG antibodies (**c**). Molecular weight markers (Mw, in kDa) are indicated on the left. Pull-down assays have been performed at least in triplicate, and a representative experiment is shown. **d** Schematic representation of the VgrG (orange), EagR (blue), and Rhs1 (red) interaction network based on pull-down experiments.

We recently showed that the *P. laumondii* Plu0353 Rhs1 protein, encoded within a Type VI secretion system (T6SS) gene cluster (Fig. 1a), is delivered by the T6SS and participates in antibacterial competition by inhibiting protein synthesis^51^. The full-length Rhs1 protein was shown to form a tripartite complex with the VgrG spike and the EagR chaperone^51^. To get further insights on the architecture of this complex, we conducted pairwise co-purification experiments using C-terminally FLAG-tagged Rhs1 (‘R’), N-terminally 6×His-tagged VgrG (‘V’) or C-terminally Strep-tagged EagR (‘E’). Figures 1b and 1c show that the full-length Rhs1 protein is unstable in the absence of the EagR chaperone (Fig. 1b, c, lanes ‘R’ and ‘V + R’), suggesting that these two proteins interact. Indeed, FLAG-tagged Rhs1 copurifies with strep-tagged EagR (Fig. 1b, c, lane ‘E + R’). By contrast, the EagR chaperone does not pull down VgrG (Fig. 1b, c, lane ‘E + V’). The tripartite complex comprising VgrG, EagR and Rhs1 (Fig. 1b, c, lane ‘E + V + R’) could be readily purified, suggesting that the EagR-stabilized Rhs protein contacts the VgrG spike. We propose a model of the architecture of the tripartite complex in which EagR-bound Rhs1 interacts with VgrG (Fig. 1d).

**Fig. 2:**
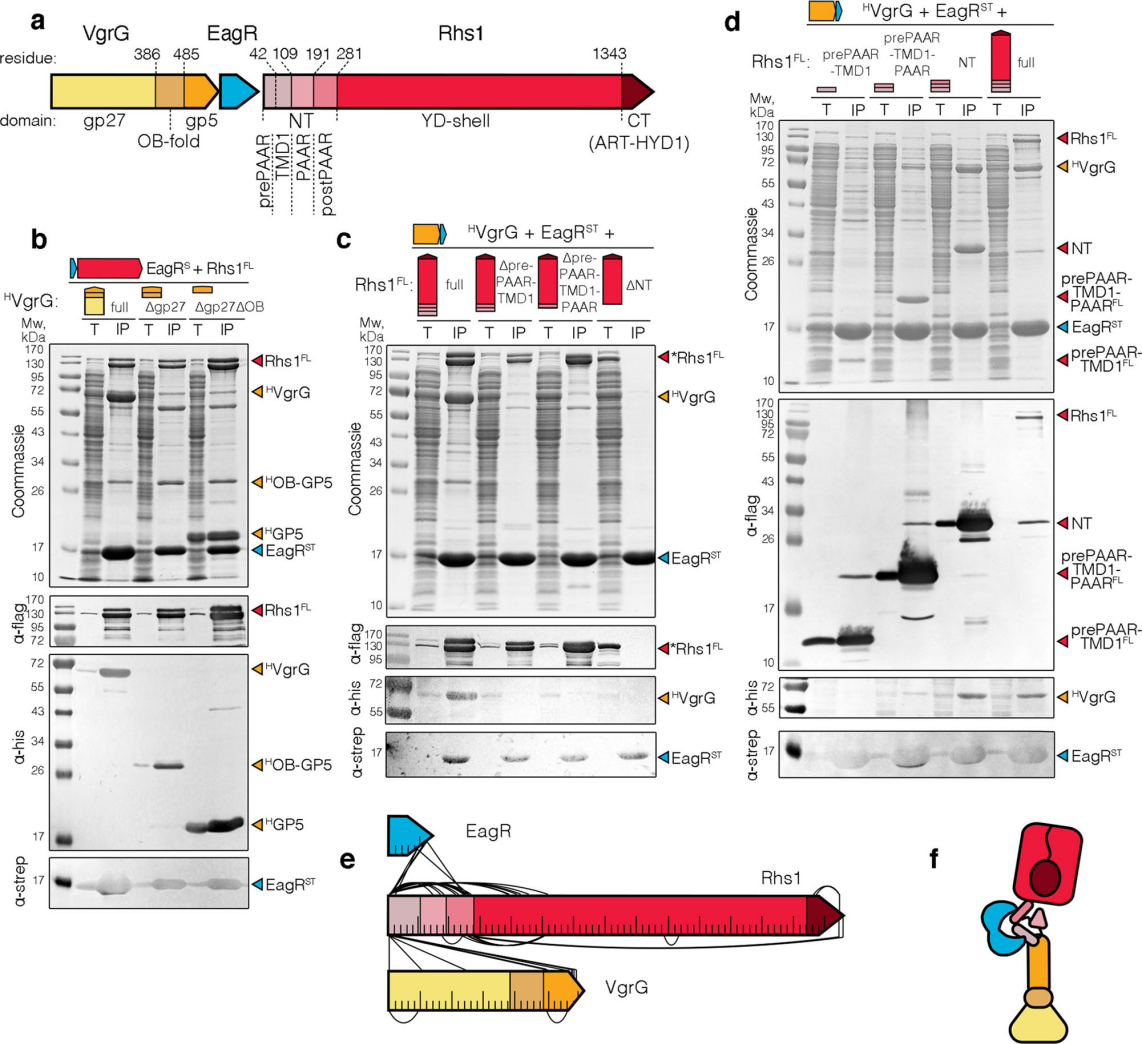
Architecture and contact map of the VgrG-Rhs1-EagR complex. **a** Schematic representations of *Photorhabdus laumondii* VgrG and Rhs1 proteins. The protein domains are indicated, as well as their boundaries. **b**–**d** Pull-down assays. Total cell extracts (T) from *E. coli* BL21(DE3) cells producing Strep-tagged EagR (^ST^EagR), FLAG-tagged Rhs1 (Rhs1^FL^) with His_6_-tagged VgrG (^H^VgrG) truncated variants (**b**) or His_6_-tagged VgrG (^H^VgrG), Strep-tagged EagR (^ST^EagR) with FLAG-tagged Rhs1 (Rhs1^FL^) truncated variants (**c** and **d**) were subjected to purification on streptactin-agarose beads. Strep-tagged and co-precipitated proteins were eluted with desthiobiotin (IP). Total and IP fractions were analyzed by SDS-PAGE and co-purified proteins were stained by Coomassie blue (upper panel) or immunodetected using anti-His, anti-Strep, and anti-FLAG antibodies (lower panels). Molecular weight markers (Mw, in kDa) are indicated on the left. Pull-down assays have been performed at least in triplicate, and a representative experiment is shown. **e** Schematic representation of intermolecular and intramolecular contacts between the EagR, Rhs1, and VgrG proteins identified by cross-link mass spectrometry. For clarity reasons, only the contacts that have minimal distance of 50 amino acids are shown. All cross-linked peptides are listed in Supplementary Table 1, and a map with all the contacts is shown as Supplementary Fig. 5. **f** Schematic model of the *Photorhabdus laumondii* VgrG (shades of orange)-EagR (blue)-Rhs1 (shades of red) complex architecture based on pull-down and cross-link mass spectrometry experiments.

To provide detailed information onto the architecture of the VgrG-Rhs1-EagR complex, we engineered truncations of the VgrG and Rhs1 proteins, and tested the ability of these truncated variants to form complexes. The *P. laumondii* Plu0355 VgrG protein shares the prototypical VgrG organization: from N- to C-terminus it comprises a gp27-like domain followed by OB-fold and gp5-like needle domains (Fig. 2a; Supplementary Fig. 1). Figure 2b shows that variants deleted of the gp27 domain only, or of the gp27 and OB-fold domains, are still capable to assemble the tripartite complex with EagR and Rhs1, demonstrating that the gp5 domain is sufficient to promote complex formation. This result suggests that the Rhs1-EagR complex binds to the tip of the needle, such as been shown for the enteroaggregative *E. coli* Tle1 phospholipase, or the *Pseudomonas aeruginosa* Tse6 effector^50,52,53^. A similar truncation approach was then performed for the Rhs1 protein. The Rhs1 protein comprises, from N- to C-terminus, a short 30-amino-acid sequence, called ‘prePAAR’ domain, followed by a predicted transmembrane domain (TMD1), a PAAR domain, a postPAAR domain comprising a conserved α-helix, the Rhs YD-shell and the Tre23 toxin domain (Fig. 2a; Supplementary Fig. 2). Pull-down of the tripartite complex with Rhs1 N-terminal truncations deleted of prePAAR, prePAAR-PAAR, or prePAAR-PAAR-postPAAR, shows that deletion of prePAAR is sufficient to prevent attachment of the EagR-Rhs1 complex to VgrG, while EagR-Rhs1 interaction is only lost when Rhs1 is missing the entire N-terminus composed of prePAAR, PAAR and postPAAR (Fig. 2c). Experiments performed with Rhs1 C-terminal truncations showed that prePAAR is sufficient to interact with EagR, while prePAAR-PAAR is sufficient to recruit VgrG to the EagR-Rhs1 complex, although the VgrG-EagR-Rhs1 complex is fully stabilized when the complete N-terminus of Rhs1 comprising prePAAR-PAAR-postPAAR is produced (Fig. 2d). Finally, the isolated postPAAR region is also co-precipitated by EagR (Supplementary Fig. 3). Taken together, the results of Rhs1 N- and C-terminal truncations suggest that prePAAR-PAAR is sufficient to interact with VgrG, while Rhs1 presents two binding sites for EagR located at TMD1 and postPAAR, respectively. In agreement with these results, and as shown for the *P. protegens* RhsA protein^21^, stability assays showed that the N-terminal region of Rhs1, comprising prePAAR-TMD1-PAAR-postPAAR, is an unstable domain that requires EagR for its stabilization (Supplementary Fig. 4). To test this model, the purified VgrG-Rhs1-EagR complex was subjected to in vitro cross-linking with NNP9, a chemical cross-linker that carries two NHS carbamate reactive groups and an azido function allowing further enrichment of cross-linked peptides through click-chemistry^54^. Mass spectrometry analysis of cross-linked peptides revealed that the three proteins establish extensive contacts (Fig. 2e; Supplementary Table 1; Supplementary Fig. 5). Notably, interactions between EagR and Rhs1 TMD1 and postPAAR were confirmed. This approach also demonstrated numerous contacts between the VgrG C-terminus and Rhs1 prePAAR, although few contacts between Rhs1 prePAAR and the gp27 base of VgrG were also identified. In addition, intramolecular contacts were revealed between the N-terminal region of the prePAAR domain and PAAR, and between the N-terminus of the Rhs1 shell and the prePAAR and postPAAR domains, as well as with the toxin domain (Fig. 2e). Taken together, these results establish a structural organization of the VgrG-Rhs1-EagR complex in which the prePAAR-PAAR N-terminus of Rhs1 sits on the tip of the VgrG gp5-like β-helix while two EagR molecules embed the Rhs1 TMD1 and postPAAR domains (Fig. 2f). This model agrees with recent data demonstrating that the *P. aeruginosa* EagR-like EagT6 chaperone interacts with the Tse6 effector TMDs, and that prePAAR and PAAR domains mediate effector-VgrG interactions^21,50^. In addition, the contacts detected between the Tre23 C-terminal toxin domain and the Rhs1 N-terminus suggest that the toxin domain is encapsulated into the Rhs1^Shell^ barrel (Fig. 2f).

### Cryo-EM structure of the Rhs1-EagR complex

**Fig. 3:**
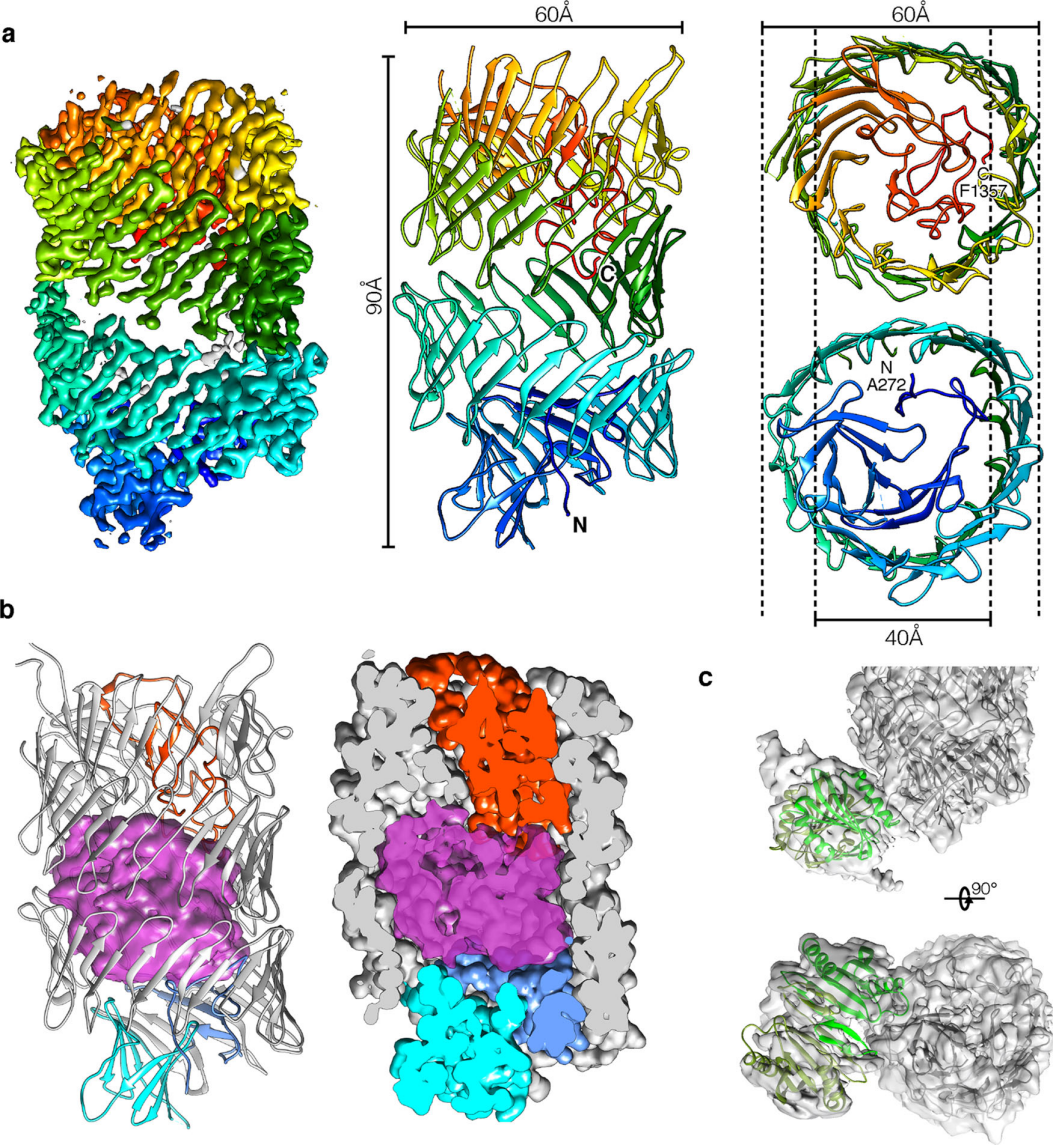
Cryo-electron microscopy structure of the Rhs1-EagR complex. **a** *P. laumondii* Rhs1 cryo-EM density map and model. Unsharpened masked cryo-EM map (left panel – 0.015 contour level) of the Rhs1 β-barrel colored accordingly to the fitted side view of the molecular model (central panel), on a blue (N-terminal) to red (C-terminal) gradient. Slices of the N-terminal (right panel-top) and C-terminal (right panel-bottom) regions are shown in a perpendicular orientation in order to highlight the plugs on each side of the β-barrel, with dotted lines depicting their inner and outer diameters and labels marking the N- and C-terminal sites with respective residue numbers. **b** Structural evidence for toxin localization inside the Rhs1 β-barrel. Amorphous low-resolution density shown in the center of the β-barrel (left), generated by subtracting all the unsharpened masked map (0.003 contour level) within 2 Å radius of the molecular model, indicated the presence of a highly flexible or unfolded sequence in the central space. By generating a 2 Å molecular density around the model (right panel), the interaction with the N- and C-terminal plugs is evidenced. The N-terminal seal and Ig-like plug domains are shown in blue and cyan, respectively, the C-terminal plug is shown in red, and the C-terminal Rhs1^CT^ toxin is shown in magenta. **c** EagR-Rhs1^NT^ interaction. Density of the N-terminal region in the unmasked map (0.004 contour level) is shown in transparency, with fitted Rhs1 (gray) and EagR dimer model (light and dark green). Only the EagR dimer model is fitted into the Rhs1^NT^-EagR density.

Detailed information about the Rhs1-EagR molecular architecture was obtained using single-particle cryo-EM. The Rhs1-EagR complex was purified from *E. coli* overproducing FLAG-tagged Rhs1 and Strep-tagged EagR by Streptag affinity followed by size-exclusion chromatography (Supplementary Fig. 6). In order to prevent Rhs1 autocleavage, a catalytic-null mutant, D1338N (see below), was used. The structure of the complex was solved using an in-house Talos Arctica 200 kV microscope equipped with a K2 summit direct detector. The 2D class averages display well-resolved views of the Rhs1 YD-shell decorated by a flexible extension (Supplementary Fig. 7a, b). Such an organization is very similar to that observed for the *P. fluorescens* RhsA-EagR complex by negative-stain electron microscopy^21^. We then performed an unmasked refinement to 3.72 Å (Supplementary Fig. 7f) followed by a local refinement on Rhs1^Shell^ and obtained a resolution of 3.17 Å for this region (Supplementary Fig. 7c, d, e; Supplementary Table 2). In the high-resolution cryo-EM densities obtained (Fig. 3a and Supplementary Fig. 7g), we could build de novo an atomic model of Rhs1^Shell^ from amino acids (aa) 272 to 1357 (Fig. 3a). Rhs1^Shell^ is composed of a continuous strip of antiparallel sheets (residues 420-1262) defining a hollow barrel, which is closed on each side by two plugs (residues 272–420 and 1263-1357 respectively) (Fig. 3a). The C-terminal plug is deeply embedded in the Rhs1 β-barrel and tightly closes one side of the barrel (Fig. 3a, b). This domain corresponds to an aspartyl protease domain that is conserved in homologous Rhs proteins and in YD-repeat proteins such as in the *Y. entomophaga* ABC toxin^34^. The N-terminal plug caps the other side of the Rhs1 β-barrel but remains at the exterior (Fig. 3a, b). It is composed of an Immunoglobulin-like (Ig-like) domain (336–421) preceded by a small extended domain sealing it to the Rhs1 β-barrel (Fig. 3a, b). The β-barrel and the two plugs define a central cavity of ~28,000 Å^3^ in volume (Fig. 3a, b). In this cavity, densities are visible but not well resolved (Fig. 3b), confirming that the C-terminal toxin domain is encapsulated into the Rhs1 β-barrel but suggesting that it is highly flexible and/or partially unfolded.

The N-terminal plug is decorated by an external highly flexible density that corresponds to the N-terminal portion of the Rhs1 protein (Rhs1^NT^) in complex with an EagR dimer (Fig. 3c). A homology model of the chaperone dimer (Supplementary Fig. 8) could be fitted in the low-resolution density obtained (Fig. 3c).

### Rhs1 autocleavage

**Fig. 4:**
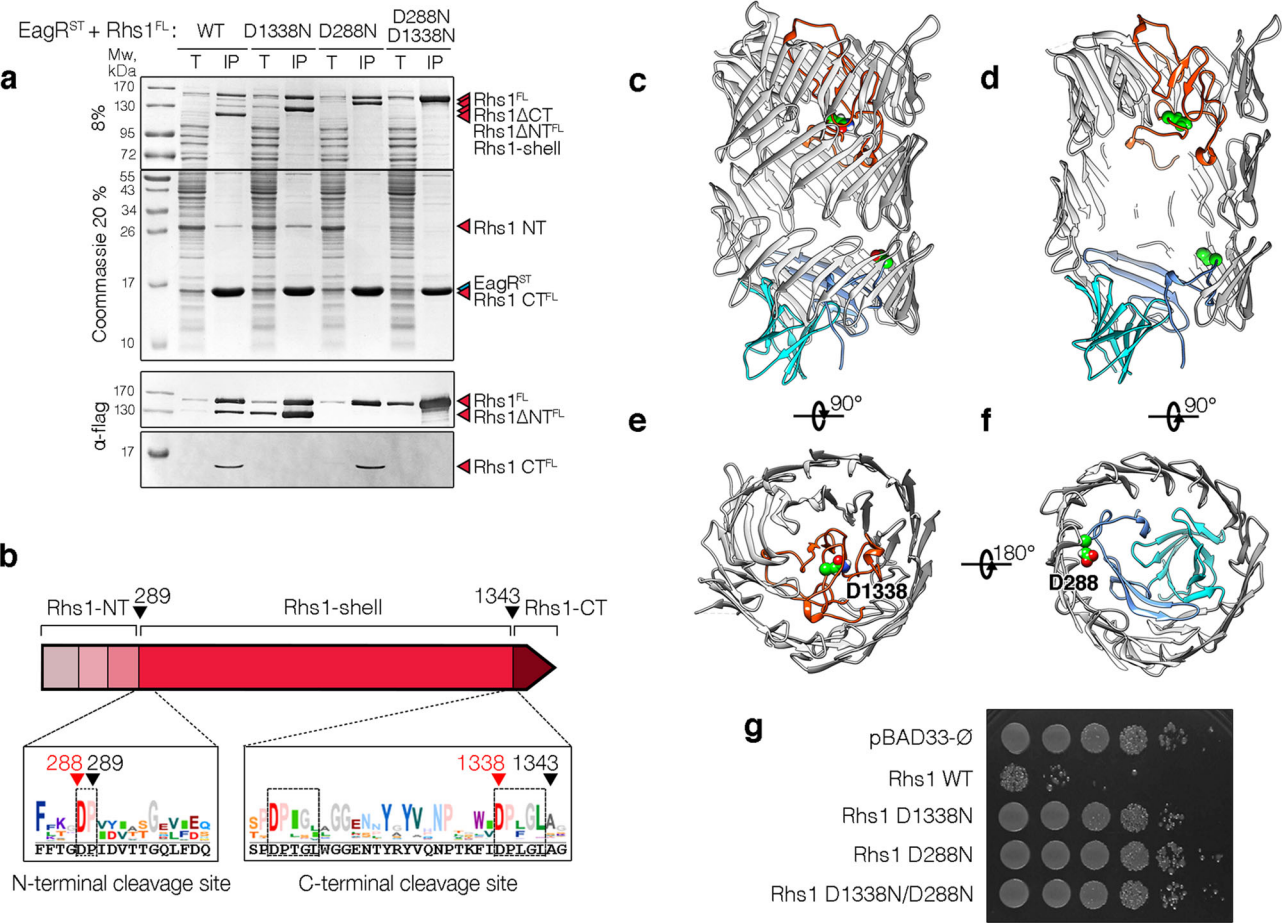
N- and C-terminal autocleavages of Photorhabdus laumondii Rhs1. **a** Pull-down assays. Total cell extracts (T) from *E. coli* BL21(DE3) cells producing Strep-tagged EagR (^ST^EagR) and the indicated D288N, D1338N and double D288N-D1338N FLAG-tagged Rhs1 (Rhs1^FL^) variants were subjected to purification on streptactin-agarose beads. Strep-tagged and co-precipitated proteins were eluted with desthiobiotin (IP). Total and IP fractions were analyzed by SDS-PAGE and co-purified proteins were stained by Coomassie blue (upper panels) or immunodetected using anti-FLAG antibodies (lower panels). The Rhs1 fragments (Rhs1ΔCT, Rhs1ΔNT, Rhs1CT and Rhs1ΔNTΔCT) are indicated on right. Molecular weight markers (Mw, in kDa) are indicated on the left. Pull-down assays have been performed at least in triplicate, and a representative experiment is shown. **b** Schematic representation of the Rhs1 protein highlighting the different domains and the sequence conservation of N- and C-terminal cleavage sites (logos). The sequences corresponding to the *P. laumondii* Rhs1 protein are shown in black, below the logos. Black arrows indicate the cleavage sites, red arrows indicate catalytic amino acid residues implicated in cleavage. **c**–**f** Position of the cleavage sites on the Rhs1 cryo-EM structure. The N- and C-terminal plugs are shown in blue and red, respectively. The aspartate catalytic residues are indicated and shown in green balls. **g** Toxicity assays. Serial dilutions (10^0^–10^−5^) of normalized cultures of *E. coli* K-12 DH5α cells carrying the empty pBAD33 vector, or pBAD33 vectors producing the full-length *P. laumondii* Rhs1 protein (Rhs1 WT) or substitution in the aspartyl catalytic residues abolishing the N-terminal (D288N), C-terminal (D1338N) or both N- and C-terminal (D1338N/D288N) cleavages were spotted on LB agar plates supplemented with 1% glucose. Toxicity assays were done in triplicate, and a representative experiment is shown.

The cryo-EM structure of the Rhs1-EagR complex shows that the Rhs1 Tre23 toxin domain is encaged inside the Rhs1 β-barrel (Fig. 3b), suggesting that drastic conformational changes or cleavage(s) should occur to release the toxin. Interestingly, the Rhs1 protein from *A. dhakensis* has recently been demonstrated to undergo two autocleavages, located at each side of the β-barrel^10^. The profile of the purified *P. laumondii* Rhs1 protein reveals four major bands corresponding to full-length Rhs1, and three cleaved products (Fig. 4a). Mass spectrometry analyses and N-terminal sequencing of these products defined that they correspond to N-terminal (Rhs1^NT^), central (Rhs1^Shell^) and C-terminal (Rhs1^CT^) fragments. The 29-kDa Rhs1^NT^ N-terminal fragment comprises prePAAR, TMD1, PAAR, postPAAR, and stops after residue Pro-289 (Fig. 4b). The ~120-kDa Rhs1^Shell^ fragment starts at Ile-290 and ends at Leu-1342 and corresponds to the β-barrel with N- and C-terminal plugs that are folded inside the first and last rings of the barrel (Fig. 4b–f). Finally, the 16-kDa Rhs1^CT^ C-terminal fragment comprises the Tre23 toxin domain that starts at residues Ala-1343 (Fig. 4b). Notably, while EagR interacts only with Rhs1^NT^ TMD1 and postPAAR, all Rhs1 fragments are pulled-down by EagR, suggesting that the Rhs1^NT^, Rhs1^Shell^ and Rhs1^CT^ fragments remain associated together after cleavage, as observed with the *A. dhakensis* Rhs protein^10^. The C-terminal cleavage site is located after the two canonical DPLGL motifs separated by 17 residues also found in Tc-toxins and *A. dhakensis* Rhs^10,55^, with the catalytic aspartate residue being part of the second motif (Fig. 4b). Indeed, substitution of the Asp-1338 residue (D1338N) abolished C-terminal cleavage (Fig. 4a). The location of the N-terminal cleavage site is less clear as no sequence corresponds to the signature of aspartyl proteases. However, a sequence alignment of the 250 best BLAST hits of Plu0353 revealed a ^289^PVYVA*S*GE signature (Fig. 4b) that shares conserved residues with the PVSMV*T*GE motif defined by Pei et al^10^. Interestingly, Pro-289 is located immediately downstream a highly conserved aspartate residue, such as in the DPLGL motif of aspartyl proteases (Fig. 4b). In agreement with these observations, the D288N mutation abolished N-terminal cleavage (Fig. 4a). Finally, the double D288N-D1338N Rhs1 variant remained intact (Fig. 4a). To test whether these cleavages are physiologically relevant to liberate the Tre23 toxin domain, the toxicity of the full-length Rhs1 protein, and of its D288N, D1338N, and D288N-D1338N variants, was assayed in *E. coli* cells. Figure 4g shows that while the full-length Rhs1 protein is highly toxic, all the cleavage variants are inactive, suggesting that the Tre23 domain needs to be released from the Rhs1 encapsulation cage to exert its lethal activity. Interestingly, in the cryo-EM map, densities are unresolved before D288 and after D1338, indicating that the Rhs1^NT^ and C-terminal toxin domains are flexible. However, even after autocleavages, it is likely that the two lid domains will remain inside the barrel, on each side of the Rhs1^CT^ toxin, such as in the *Y. entomophaga* ABC toxin^34^. Further events will therefore be needed to liberate the toxin domain.

## Discussion

In this work, we report the architecture and structure of a Rhs polymorphic toxin associated with the type VI secretion system in *P. laumondii*. We show that Rhs1 is composed of three main regions: a Rhs1^NT^ N-terminal region that comprises a PAAR domain followed by a transmembrane segment, a PAAR domain, and a postPAAR domain comprising a conserved α-helix, a Rhs1^Shell^ central domain that folds as a β-barrel closed by plugs on each side, and a Rhs1^CT^ C-terminal domain that comprises the Tre23 ADP-ribosyltransferase toxin activity. The EagR chaperone binds Rhs1^NT^, specifically at the level of TMD1 and postPAAR. Rhs1^NT^. The prePAAR and PAAR domains are involved in the interaction with the β-needle tip of the VgrG transporter protein. The Tre23 toxin domain is encapsulated inside the β-barrel. We also demonstrate that Rhs1 undergoes two aspartate-catalyzed self-cleavages located at both sides of the Rhs1^Shell^.

### A model for T6SS-dependent delivery of Rhs toxins

**Fig. 5:**
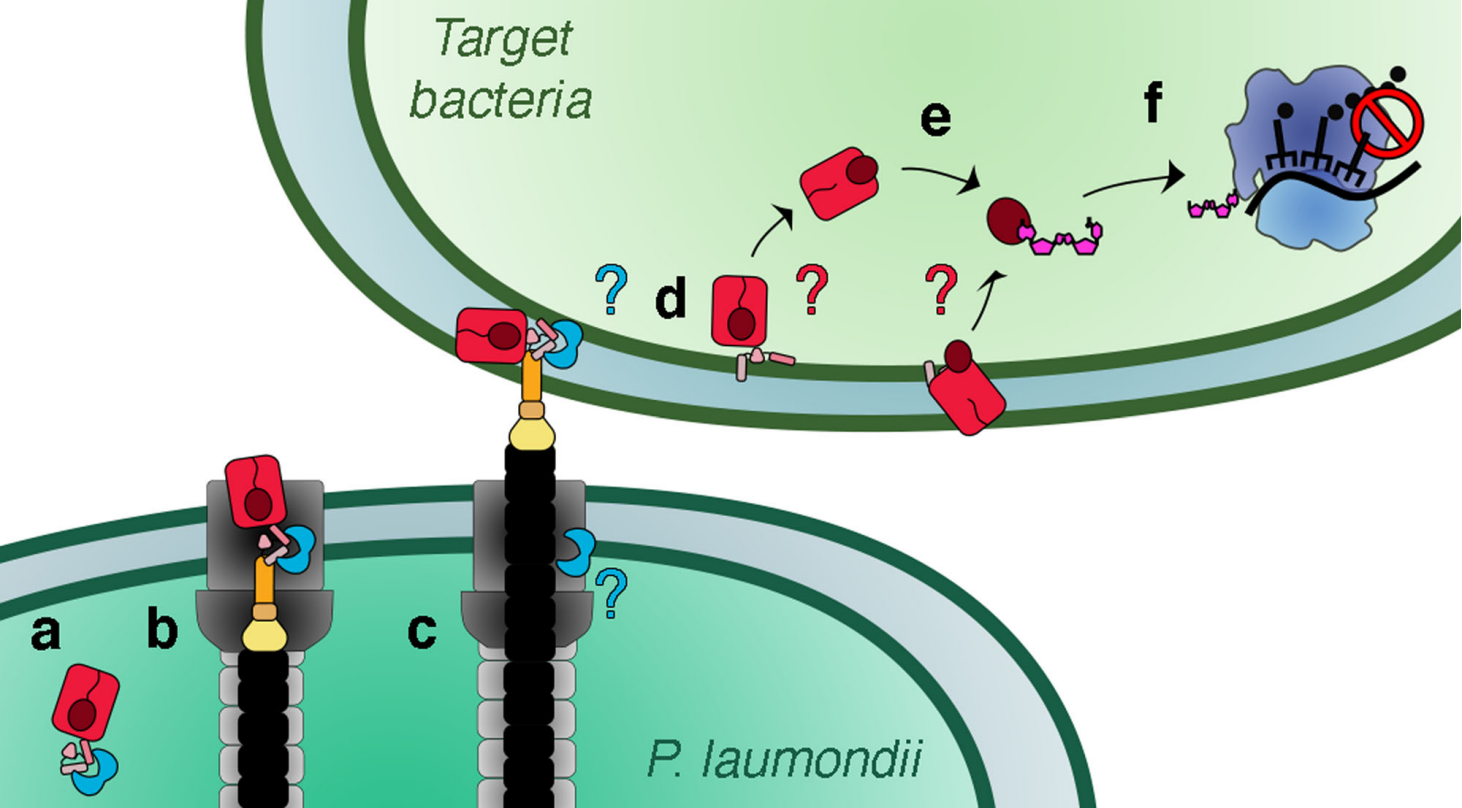
Model for Rhs loading and delivery by the type VI secretion system. **a** The EagR chaperone (blue) binds to the Rhs^NT^ hydrophobic domains to stabilize the Rhs protein (red) prior loading on the T6SS. **b** The Rhs protein is loaded on the C-terminus of the VgrG spike (orange) via its N-terminal prePAAR-PAAR domain. **c** Sheath contraction propels the inner tube (black) to inject the VgrG loaded with Rhs into the target cell. It is not known whether the EagR chaperone remains in the attacker cell or whether it is translocated together with the VgrG-Rhs complex. **d** The Rhs1 protein is directly delivered in the cytoplasm, or if delivered in the periplasm, the Rhs^NT^ hydrophobic transmembrane domains insert into the inner membrane of the target cell and are used to translocate the Rhs β-barrel and/or the toxic domain into the cytoplasm of the target cell. Alternatively, the Rhs β-barrel itself inserts into the membrane. **e** N- and C-terminal autoproteolysis opens the Rhs shell and releases the Rhs^CT^ toxin into the target cell cytoplasm. **f** The *P. laumondii* Rhs1 C-terminal toxin domain, Tre23, arrests translation by ADP-ribosylation of the 23 S rRNA.

While the mode of translocation of Rhs toxins into recipient cells remains enigmatic, we propose a model (Fig. 5) in which the PAAR-containing Rhs protein is stabilized in the cytoplasm of the attacker cell through interactions with its cognate EagR chaperone (Fig. 5a). Upon assembly of the T6SS, Rhs is loaded onto the VgrG spike through contacts between the Rhs prePAAR-PAAR region and the tip of the VgrG gp5-like needle (Fig. 5b). In this conformation, the attacker cell is protected from the action of the Tre23 toxin by both its cognate Tri23 cytoplasmic immunity^51^ and by the β-barrel cage that encapsulates Tre23. Such a double-lock mechanism has been already demonstrated for the EAEC Tle1 phospholipase active site, which is blocked by the Tli1 immunity protein^52^, and by steric hindrance when loaded onto the VgrG spike^53^. Whether the EagR chaperone remains associated on the tip complex or dissociates after Rhs loading on VgrG is yet to be experimentally determined. Contraction of the T6SS sheath fires the complex into the recipient target cell, where it is delivered into the cytoplasm or periplasm (Fig. 5c). If the Rhs complex is delivered into the periplasm, we propose that it dissociates from VgrG and that the Rhs^NT^ TMD inserts into the inner membrane to translocate the Rhs^Shell^ β-barrel across the inner membrane (Fig. 5d). Autocleavages at the N- and C-termini of the β-barrel liberates the toxin into the cytoplasm (Fig. 5e) where it can target 23 S rRNAs to inhibit translation (Fig. 5f). Alternatively, the Rhs cage can be opened into the periplasm, and the released Tre23 C-terminal domain can be translocated through a channel formed by the Rhs TMD, or by an inner membrane protein/complex of the host. Many of these steps are hypothetical and should be experimentally addressed, *e.g*., does the EagR chaperone interact with Rhs prior to its loading onto the T6SS? Is the EagR chaperone dissociated prior secretion or in the target cell? Is the Rhs protein delivered into the periplasm or cytoplasm of the target cell? Does the Rhs β-barrel translocate across the inner membrane, insert into it or does it interact with an inner membrane protein/complex? How does the Rhs-EagR complex dissociate from VgrG? How is the toxin released or extracted from the Rhs^Shell^ cage?

### Rhs structure

We have shown that the Rhs1 central region folds as a β-barrel structure in which the two extremities are obstructed by plug domains. Interestingly, it shares the same modular architecture as Tc-toxins and eukaryotic teneurins that protect their effector domains within a barrel that defines a shell-like compartment (Supplementary Fig. 9). In Tc-toxins and Rhs, this β-barrel maintains the toxin domain confined in a closed environment and most probably maintains it partially unfolded to prevent its action in the producing cell. The volume of the Rhs1 β-barrel, ~28,000 Å^3^, is compatible with the accommodation of a <25 kDa folded domain, in agreement with the observation that Rhs C-terminal toxin domains have sizes ranging from 9 to 20 kDa (Supplementary Fig. 10). By contrast, the C-terminal domain of teneurins is exposed at the surface of the shell^31,35^ (Supplementary Fig. 9). The architectures of Rhs1, Tc-toxins, and teneurins diverge at the level of the N-terminal plug (Supplementary Fig. 9). In Tc-toxins, this plug is made of another protein that extends the β-barrel and closes it. It also contains a β-propeller domain that is essential for the opening of the shell to extract the toxin. In teneurins, the N-terminal part of the protein forms a plug made of two Ig-like domains and a β-propeller domain. The T6SS-associated Rhs proteins therefore define another class with a N-terminal plug made of an Ig-like domain and a seal domain (Supplementary Fig. 9).

### Mounting Rhs on the T6SS needle

As previously evidenced for the *S. marcescens*, *E. cloacae*, and *P. fluorescens* Rhs proteins^20–22^, we have shown in this work that *P. laumondii* Rhs1 assembles a tripartite complex with the EagR chaperone and the VgrG spike. Rhs1 is thus likely transported as a VgrG cargo, such as other effectors including the *P. aeruginosa* Tse6 PAAR-effector or the enteroaggregative *E. coli* Tle1 phospholipase^50,52,53^. However, the mode of interaction with the spike is likely to be different. While Tle1 associates to a C-terminal extension of the VgrG spike^52,53^, our truncation studies demonstrate that Rhs1-VgrG complex formation requires the EagR chaperone for Rhs1^NT^ stabilization, and the Rhs1^NT^ and VgrG gp5-like β-prism. The result agrees with the observation that deletion of the Rhs N-terminal region in *A. dhakensis* abolishes Rhs secretion^10^. Further delineation showed that the prePAAR and PAAR regions of Rhs1^NT^ are sufficient to support the interaction with VgrG. This result differs from the *A. dhakensis* VgrG-Rhs complex that requires both Rhs^NT^ and Rhs^CT^ for efficient interaction^10^ but is identical to the formation of the *P. protegens* VgrG1-RhsA complex that only requires Rhs^NT ^^21^. Negative-stain electron microscopy analyses of the *P. protegens* VgrG1-RhsA-EagR1 complex showed that the prePAAR-TMD-PAAR region of RhsA mediates contacts with the VgrG tip^21^. Based on structure modeling, Ahmad et al. suggested that the Tse6 and RhsA PAAR domains are not complete, and that prePAAR complements the PAAR fold, explaining why prePAAR is important for VgrG-RhsA or VgrG-Tse6 complex formation^21^. The Tse6 prePAAR-PAAR model shows that the His residue of the prePAAR AARxxDxxxH motif is properly positioned to participate to the tetrahedrally coordination of the Zn^2+^ atom^21^. The *P. laumondii* Rhs1 prePAAR region also bears a PAAARxxDxxxH motif that comprises the PAAR/PAAAR signature and the coordinating His residue (Supplementary Fig. 2c). Interestingly, our XL-MS data fully agree with this model as several contacts between Rhs prePAAR and PAAR were observed (Fig. 2e). Taken together, the current model proposes that the Rhs1^NT^ prePAAR and PAAR regions form a bona fide PAAR conical structure that sits on the VgrG spike, such as canonical PAAR subunits, hence anchoring Rhs1 to the tip of the T6SS needle. While the position of Rhs1 on the needle is now better defined, it remains to be determined whether Rhs1 is loaded onto the VgrG spike during T6SS biogenesis. Recent information on the *Enterobacter cloacae* T6SS have shown that Rhs is required for assembly of the apparatus^22^. However, whether Rhs interact with VgrG before its incorporation into the baseplate, or whether it is recruited to the fully assembled baseplate remains to be determined.

### The EagR chaperone and the Rhs1 TMD

Previous pull-down and two-hybrid studies showed that the EagR chaperone interacts with Rhs, and more specifically with Rhs^NT^ in *S. marcescens*, *E. cloacae*, *P. fluorescens*, and *S. enterica* Typhimurium^20–22^. Eag chaperones are required for the secretion and stability of PAAR domain-containing effectors such as *P. aeruginosa* Tse6, *P. fluorescens* Tne2 and *S. marcescens* and *P. protegens* RhsA^19–21,56,57^. Similar to our observation, Tse6 copurifies with EagT6 when individual TMDs are deleted, but not when both TMDs are deleted^50^, suggesting that Eag chaperones bind to both TMDs of PAAR-containing T6SS effectors. Indeed, the low-resolution electron microscopy structure of the VgrG-Tse6-EagT6 complex showed that two EagT6 dimers wrap the TMDs of the Tse6 effector^50^. The recent crystal structures of Eag chaperones from *P. aeruginosa* and *S. enterica* Typhimurium demonstrated that the Eag dimer has an overall horseshoe-like shape that embraces the Rhs^NT^ TMD helix bundle in a claw-like manner^21^. Binding of the Eag dimer to the Rhs^NT^ helix bundle is mediated by a combination of knob-hole hydrophobic interaction and hydrophilic residues via bifurcated hydrogen bonds^21^. A structural model of the *P. laumondii* EagR dimer shows that EagR has an identical horseshoe-like fold with a central hydrophobic cleft that can accommodate the Rhs TMDs (Supplementary Fig. 8a–c). Indeed, mapping of the Rhs cross-link contacts on the *P. laumondii* EagR dimer model shows that Rhs TMD1 and the conserved α-helix of postPAAR interact with the concave face of EagR (Supplementary Fig. 8d). Eag chaperones therefore protect the hydrophobic regions of Rhs^NT^ and other PAAR-containing effectors to prevent aggregation or premature insertion into membranes. Interestingly, Tse6 spontaneously inserts into lipid vesicles causing EagT6 chaperones to be released suggesting that EagT6 maintains the N-terminal TMDs in a preinsertion state until it reaches the inner membrane of the target cell^50^. However, this model is challenged by a study that has shown that the EagR protein is not detected in *S. marcescens* cell supernatant, contrarily to its cognate Rhs protein, suggesting that the EagR-Rhs interaction is not maintained after loading onto the T6SS apparatus^20^.

### Autocleavage and toxin release

PrePAAR-containing effectors usually being cytoplasmic-acting toxins^19,21^, the TMDs are proposed to insert into the inner membrane to promote entry of the toxin domain in the cytoplasm. In the case of Rhs effectors, the toxin domain is encapsulated into the Rhs β-barrel and hence needs to be released upon translocation. Such as *E. cloacae* and *A. dhakensis* Rhs^10,22^, the *P. laumondii* Rhs1 protein undergoes two aspartyl autoproteolysis, yielding three fragments corresponding to Rhs1^NT^, Rhs1^Shell^ and the Rhs1^CT^ toxin. Autocleavage likely occurs when the Rhs protein reaches the target cell, as no autocleavage was detected inside *E. cloacae*^22^, suggesting that certain conditions associated with the periplasm and/or inner membrane of the target activate the intramolecular aspartyl protease activity. Interestingly, TcC polymorphic toxins also undergo autocleavages^34,36,55^, and some teneurins carry C-terminal sequences that resemble neuroendocrine signaling peptides adjacently located to predicted furin cleavage sites, suggesting that the C-terminal peptides are released^58^. As shown for insecticidal TcC proteins^34,36,55^, our results demonstrated that substitutions preventing N- or C-terminal cleavage significantly reduced the toxic activity of Rhs1, and hence that autocleavages are required to liberate the Rhs1^CT^ domain in order to exert its toxin activity. In *A. dhakensis*, mutations blocking Rhs autocleavage do not impact T6SS-dependent secretion but reduces toxicity^10^. However, we and others have shown that the three fragments remain in complex upon autocleavage as they can be all pulled-down by the EagR chaperone that only binds to Rhs^NT ^^10,22^. In our conditions, and despite the two autocleavages, the Tre23 toxin is not fully liberated from the β-shell. These observations agree with our Rhs1 structure, which shows that the encapsulated Rhs1^CT^ is blocked into the Rhs1 cage by two plug regions that prevent its release from the β-barrel. While additional experiments are required to better understand how the toxin domain is liberated from the Rhs cage, one can hypothesize that specific conditions or factors in the target cell facilitate the separation of Rhs^CT^ from Rhs^Shell^, or that large conformational changes to eject the lids out of the Rhs^Shell^ or to laterally open the β-barrel shell, occur to release the Rhs^CT^ toxin.

## Methods

### Bacterial strains, growth conditions, media, chemicals, and antibodies

Bacterial strains, plasmids, and oligonucleotides used in this study are listed in Supplementary Table 3. *E. coli* DH5α strain was used for cloning and *E. coli* BL21(DE3) for protein production and purification. *P. luminescens* subsp. *laumondii* TTO1 DNA was used as the template for cloning. Bacteria were grown in Lysogeny Broth (LB), with agitation at 37 °C. When required, media were supplemented with ampicillin (100 μg mL^−1^), streptomycin (100 μg mL^−1^) or kanamycin (50 μg mL^−1^). Gene expression from pET vectors derivatives was induced by the addition 500 μM isopropyl β-D-1-thiogalactopyranoside (IPTG). SDS-PAGE, Western-blots and immunodetection have been performed using standard procedures. Gels were stained using Instant*Blue*^TM^ (Sigma-Aldrich) or transferred onto nitrocellulose membrane, and immunodetected with His-Tag (clone 1B7G5, Proteintech catalogue #66005-1-Ig, dilution 1/5,000), Strep-Tag Classic (clone Strep-tag II, Bio-Rad catalogue #MCA2489, dilution 1/1,000) or anti-FLAG (clone M2, Sigma-Aldrich catalogue #F3165, dilution 1/1,000) monoclonal antibodies, and secondary goat anti-mouse antibodies conjugated to the Alkaline Phosphatase (AffiniPure, Jackson ImmunoResearch catalogue #115-055-003, dilution 1/5,000), and revealed using 5-bromo-4-chloro-3-indolyl phosphate/nitro blue tetrazolium (BCIP/NBT) in presence of 10 mM MgCl_2_ in alkaline buffer (pH 9).

### Plasmid construction

pET derivatives plasmides producing N-terminally 6×His-TEV-tagged VgrG (pET-His-VgrG), C-terminally Strep-tagged EagR (pRSF-EagR-ST) and C-terminally FLAG-tagged Rhs1 (pCDF-Rhs-FL) have been previously described^51^. The sequences corresponding to the VgrG and Rhs1 truncated derivatives were cloned by restriction-ligation into pET-Duet^TM^-1, and pCDF-Duet^TM^-1. The sequence corresponding to full-length Rhs1 was cloned by restriction-ligation into pBAD33-rbs, a pBAD33 vector derivative in which a consensus ribosome binding site has been cloned. Briefly, *vgrG* and *rhs* DNA fragments were amplified from *P. laumondii* DNA using the Q5 Taq polymerase (NEB) and oligonucleotides inserting restriction sites. The PCR fragments were purified using the NucleoSpin Gel and PCR Clean-up kit (Macherey-Nagel) before and after digestion with BmtI and HindIII (pET-His-VgrG derivatives) or SacI-SalI (pCDF-Rhs-FL derivatives, pBAD33-rbs derivatives). Digested fragments and plasmids were then ligated with T4 DNA ligase (NEB) and transformed into *E. coli* DH5α cells. To prevent toxicity, substitutions of Tre23 toxin catalytic residues, Y1351 and Y1375^51^ were introduced by site-directed mutagenesis using complementary pairs of oligonucleotides bearing the desired mutation. PCR-amplified fragments were then purified, phosphorylated by T4PNK (NEB), ligated with T4 DNA ligase and transformed into *E. coli* DH5α cells. Clones were verified by colony-PCR, plasmids were isolated using Wizard Plus SV Minipreps kit (Promega) and their sequences were checked by DNA sequencing (Eurofins).

### Protein and complex purification

VgrG-Rhs1-EagR complex purification was done as previously described^51^. Briefly, *E. coli* BL21(DE3) cells freshly transformed with derivatives of pET-His-VgrG, pRSF-EagR-FL, and pCDF-Rhs-ST or with empty cloning vectors to yield strains with the same combination of antibiotic resistance were grown overnight and diluted 1/100 into 50 mL of LB supplemented with ampicillin, kanamycin and streptomycin. Expression of *vgrG*, *eagR*, and *rhs1* was induced at *A*_600_ = 0.8 with 0.5 mM IPTG for 18 h at 16 °C. Cells were collected by centrifugation at 4,000 × *g* and resuspended in buffer A (50 mM Tris-HCl pH8.5, 250 mM NaCl, 1 mM TCEP, cOmplete^TM^ protease inhibitor cocktail (Sigma-Aldrich)). Cells were broken by sonication on ice, and a cleared cell extract was obtained by centrifugation for 45 min at 20,000 × *g*. One hundred μL of Strep-Tactin Superflow resin (IBA Technology) equilibrated in buffer A was then added into the cell extract. After incubation for 1 h at 4 °C with gentle mixing, the resin was washed 5 times with 300 μL of buffer A, and proteins were eluted with 100 μL of elution buffer (buffer A supplemented with 2.5 mM desthiobiotin). Ten μL of 10× diluted protein extract and 7.5 μL of elution material was separated by SDS-PAGE and proteins were stained with Instant*Blue*^TM^ (Sigma-Aldrich) or transferred onto nitrocellulose membrane. Western blots were performed by standard procedures, in PBS supplemented with 0.1% Tween-20 and 3% of Bovine Serum Albumin. Tagged proteins were detected with commercial monoclonal His-Tag (clone 1B7G5, Proteintech catalogue #66005-1-Ig), Strep-Tag Classic (clone Strep-tag II, Bio-Rad catalogue #MCA2489) or anti-FLAG (clone M2, Sigma-Aldrich catalogue #F3165) antibodies, and secondary antibodies coupled to the Alkaline Phosphate, and revealed using 5-bromo-4-chloro-3-indolyl phosphate/nitro blue tetrazolium (BCIP/NBT) in presence of 10 mM MgCl_2_ in alkaline buffer (pH 9).

### Cross-linking mass spectrometry

#### Sample preparation and cross linking

The VgrG-Rhs1-EagR complex was purified from a 100-mL culture as detailed above, with the exception that buffer B (50 mM HEPES pH7.5, 250 mM NaCl, 1 mM TCEP, cOmplete^TM^ protease inhibitor cocktail) was used instead of buffer A to avoid the Tris buffer that interferes with chemical cross-linking. One hundred nmoles of the NNP9 crosslinker^54^ were added to 200 µg of the complex. After incubation for 30 min at 4 °C, the reaction was quenched by the addition of 50 mM ammonium bicarbonate for 15 min at 4 °C. Samples were flash frozen in liquid nitrogen until further analyses. Protein digestion, cross-linked peptides enrichment, and mass spectrometry analysis were performed as previously described^59^.

#### Protein digestion

Cross-linked proteins were transferred onto a 0.5-mL Amicon 30 kDa filter, and the excess of cross-linker was removed by six concentration-dilution cycles with 50 mM ammonium bicarbonate. Proteins were digested overnight on the filter, with a trypsin:protein ratio of 1:100 (w:w). Tryptic peptides were recovered from the filter after centrifugation.

#### Enrichment of labeled peptides

Peptides were purified by click-chemistry on a photocleavable support. Photo-cleavable alkyne (PCA) agarose beads (1 µL, 10 nmol) were mixed with the tryptic peptides, in presence of copper(II) sulfate (5:1), Tris(benzyltriazolylmethyl)amine (THPTA; 25:1) and sodium ascorbate (50:1) (ratios are given relative to NNP9 concentration). After 30 min of at room temperature with gentle agitation, PCA agarose beads were washed four times with PBS and twice with 0.1% formic acid to remove unlabeled peptides. PCA agarose beads were transferred onto a 96-well plate (10 µL PCA agarose beads maximum per well), covered with 50 µL of 0.1% formic acid and placed 10 min under UV and rotational mixing (500 rpm). Released peptides were pipetted and transferred into the polypropylene injection vial.

#### Cross-linked peptides MS analysis

Peptides eluted from PCA agarose beads were analyzed by nanoLC-MS/MS using an EASY-nLC™ 1200 system (Thermo-Scientific) coupled to the nanoelectrospray ion source of an Orbitrap Q Exactive HF mass spectrometer (Thermo-Scientific). Peptides were loaded on an in-house packed nano-HPLC column (75 µm × 25 cm) containing C_18_ resin (Aeris PEPTIDE XB-C18, 1.7-μm particles, 100-Å pore size, Phenomenex) and separated by reverse-phase chromatography at 250 nL/min. A 1-h linear gradient from 8 to 30% solvent B (80% acetonitrile, 0.1% formic acid) followed by a 35 min ramp up to 60% solvent B was used. The Orbitrap mass spectrometer was set up in data-dependent acquisition mode. After a survey scan over the 300–1500 m/z range in the Orbitrap (resolution 60k at m/z 200), the 10 most intense precursor ions above 4.2 × 10^5^ intensity with 3 to 8 charges were selected for HCD fragmentation with a normalized collision energy (NCE) set up to 26.

#### Data analysis

All raw data were processed using MassSpec Studio V2.4^60^. Data were analyzed using the “E-Value Generator” algorithm with PercentEValueThreshold set at 0.1. Cross-linked peptide identifications were confirmed manually and discrepancies in the cross-linked sites were resolved to ensure that 2 chromatographic peaks did not share the same pair of sites.

### Purification of EagR-Rhs1 complex for structural studies

*E. coli* BL21(DE3) cells freshly transformed with pRSF-EagR-ST and pCDF-Rhs-FL carrying the D1338N, Y1351A and Y1375A mutations to avoid autocleavage and toxicity were grown overnight in LB and diluted 1/100 into 4 L of LB supplemented with kanamycin and streptomycin. At *A*_600_ = 0.8, expression of *eagR* and *rhs1* was induced by the addition of 0.5 mM IPTG for 18 h at 16 °C. Cells were collected by centrifugation at 4,000 × *g*, resuspended in buffer A, and broken using an Emulsiflex-C5 (Avestin). The protein extract was cleared by centrifugation for 30 min at 20,000 × *g*, filtered through a 0.45 μM membrane (ClearLine) and loaded on a 1-mL StrepTrap HP column (Cytiva) equilibrated in buffer A. The column was washed with 20 mL of buffer A and proteins were eluted with 10 mL of buffer A supplemented with 2.5 mM of desthiobiotin. Peak fractions of highest concentrations were pooled and loaded on a Superose 6 10/300 column (Cytiva) equilibrated with 50 mM Tris-HCl pH8.5, 250 mM NaCl. The EagR^ST^-Rhs1^FL^ complex eluted as a single monodisperse peak and was separated from the EagR^ST^ excess.

### Cryo-EM sample preparation, data collection, and processing

#### Sample preparation

Quantifoil Cu300 mesh R2/2 grids were glow discharged at 0.3 mbar vacuum and 2 mA current for 35 s. Then, 4 µL of purified protein complex was added to the grid and blotted for 1.5 s in a Vitroblot instrument (FEI), at 100% humidity and 4 °C, at blot force 0, before being plunge-frozen in liquid ethane and transferred/stored in liquid nitrogen.

#### Data collection and processing

Data collection and processing flowchart are summarized in Supplementary Fig. 11. Data collection was performed in a 200 kV Talos Arctica (FEI) equipped with a K2 camera (Gatan) at 45k magnification, pixel size of 0.93 Å, total dose of 50 e-/Å^2^ and 3.7 s exposition, using SerialEM version 3.8. A total of 8550 movies were collected and processed using the Relion 3.1 software^61^. Two thousand particles were manually picked and submitted to 2D classification in order to generate initial templates, which were in turn used for template-based picking. 2,623,687 particles were extracted with a 280-pixel box size resized to 96 pixel and submitted to several rounds of 2D classification, resulting in 1,036,869 selected particles, which were re-extracted with a box size of 280 pixels with original pixel size. A global 3D refinement resulted in a 3.72 Å resolution map. The refinement was repeated with a mask on the Rhs1 barrel region, which led to a 3.46 Å consensus map. After per-particle CTF refinement and Bayesian polishing, this same dataset retrieved a 3.1 Å map, but in which the C-terminal region of the Rhs1 barrel was suboptimally defined. This map was used for a 3D classification without sample alignment using a mask on the Rhs1 barrel and a separation in eight classes, which retrieved two major classes and six classes containing poorly-aligned particles. The best-defined class was selected, containing 140,577 particles, which led to the final map at 3.17 Å upon masked refinement. The final map was sharpened using the Autosharpen tool from the Phenix package^62^ using the two half maps as input. De novo model building was performed by tracing the main chain manually in Coot^63^ and submitting it to the map-to-model tool from the Phenix package, followed by manual building in Coot, optimization in ISOLDE, and finished using the Real-Space-Refine tool from the Phenix package. Model validation was analyzed in Coot and then verified using the Comprehensive validation tool from the Phenix package.

### Bioinformatic analyses and modeling

Sequence conservation of Rhs1-like proteins was estimated by BlastP and sequence alignment was made with standard algorithm parameters (NCBI). The alignment was then treated with JalView 2.11.1.4 to generate a consensus sequence logo. Transmembrane segments were predicted using TMHMM server v.2.0^64^ and hydrophobicity was analyzed by ProtScale (Expasy) using Kyte & Doolittle scale^65^. VgrG and EagR 3D models were obtained using Galaxy Homomer^66^ tool on GalaxyWEB server using the *P. aeruginosa* VgrG1 (PDB: 4MTK; unpublished) and EagR (PDB: 1TU1; unpublished) structures as templates, and by imposing trimeric (VgrG) and dimeric (EagR) oligomeric states. Rhs1^NT^ model was made using AphaFold2^67^. Structures were visualized and figures were prepared using Chimera^68^.

### Reporting Summary

Further information on research design is available in the Nature Research Reporting Summary linked to this article.

## Supplementary information

Supplementary Information

Reporting summary

## Data Availability

The final cryo-EM density map of the *P. laumondii* Rhs1-EagR complex has been deposited to the Electron Microscopy Data Bank (EMDB) under the accession code EMD-13587. The final atomic model was deposited into the Protein Data Bank (PDB) under the accession code 7PQ5. The proteomics data have been deposited to the ProteomeXchange Consortium via the PRIDE partner repository with the dataset identifier PXD028652. The data are provided in the Supplementary Information files or can be obtained from the corresponding authors upon request. Uncropped gels and Western-blots are shown in Supplementary Fig. 12.
