# Supplementary material for: Mounting, structure and autocleavage of a type VI secretion-associated Rhs polymorphic toxin
Source: Nat Commun. 2021 Dec 1;12:6998. doi: 10.1038/s41467-021-27388-0 (PMC8636562; doi:10.1038/s41467-021-27388-0)
Supplement: Supplementary file 1 — Supplementary Information [file 41467_2021_27388_MOESM1_ESM.pdf]

SUPPLEMENTARY INFORMATION

Mounting, structure and autocleavage of a type VI secretion-associated Rhs polymorphic toxin

Dukas Jurėnas, Leonardo Talachia Rosa, Martial Rey, Julia Chamot-Rooke, Rémi Fronzes, Eric Cascales

Supplementary Figures and Captions

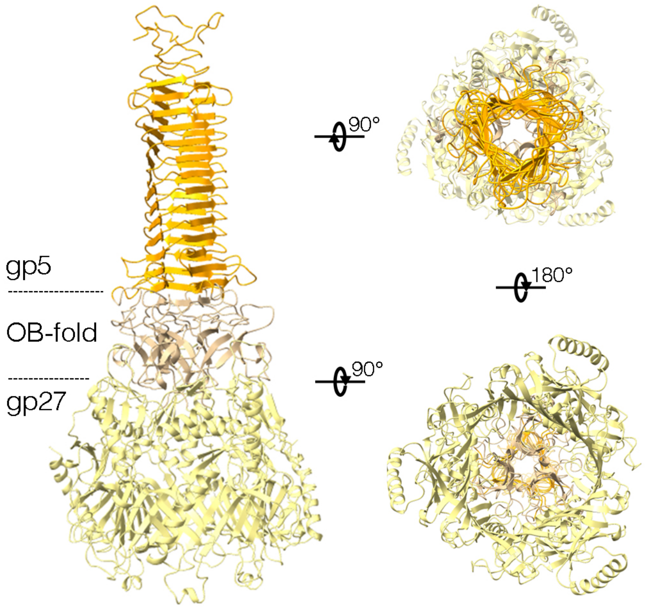

Supplementary Figure 1 | *P. laumondii* VgrG structural model. The model was generated using the Galaxy Homomer server and the *P. aeruginosa* VgrG1 protein (PDB: 4MTK) as template, imposing a trimeric oligomeric state. The different VgrG domains are indicated, and colored as used throughout the article's figures.

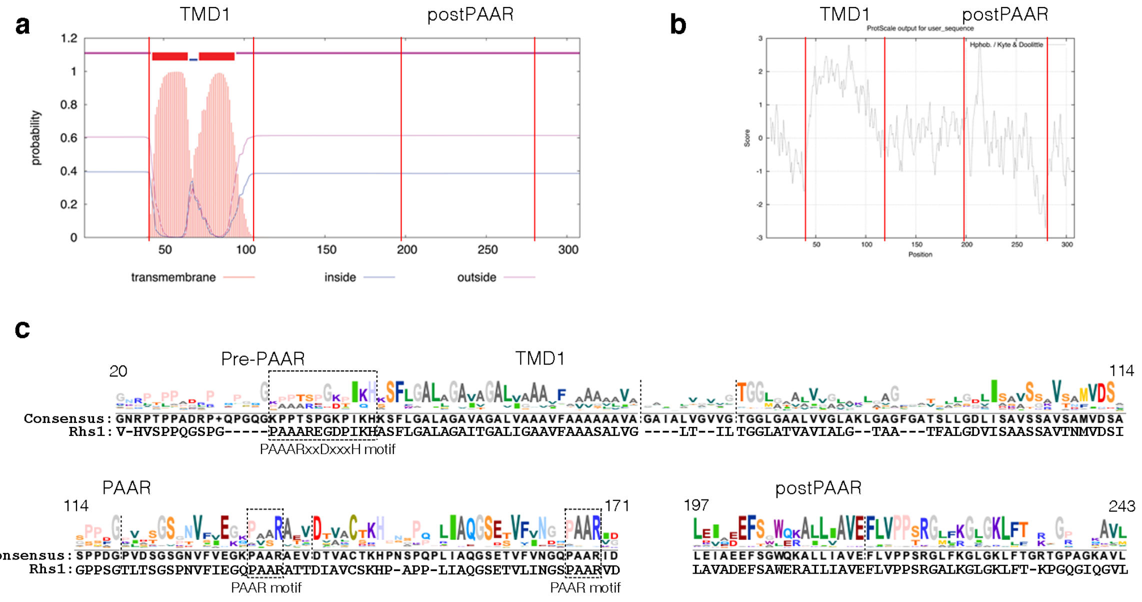

Supplementary Figure 2 | Hydrophobicity and conservation of the Rhs1^{NT} domain. (a) Transmembrane segments prediction using the TMHMM server. (b) ProtScale (Expasy) hydrophobicity analysis using Kyte & Doolittle scale. (c) Sequence logo highlighting the residue conservation in the prePAAR, TMD1, PAAR and postPAAR domains. The sequence logo was generated from an alignment of 250 homologous sequences. Conserved PAAR motifs are boxed, conservation consensus and *Photorhabdus* Rhs protein sequence are provided below.

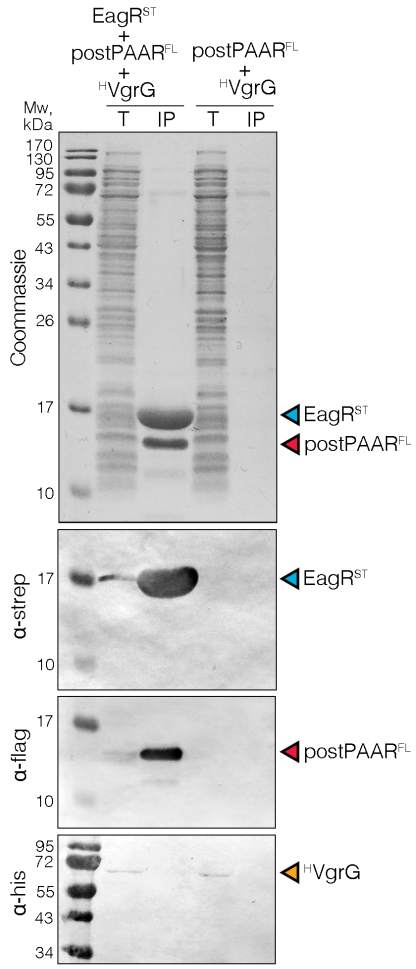

Supplementary Figure 3 | EagR binds to the Rhs1 postPAAR region. Pull-down assay. Total cell extracts (T) from *E. coli* BL21(DE3) cells producing His_6-tagged VgrG (^{H}VgrG) and FLAG-tagged Rhs1-postPAAR in presence or absence of Strep-tagged EagR (STEagR), were subjected to purification on streptactin-agarose beads. Strep-tagged and co-precipitated proteins were eluted with desthiobiotin (IP). Total and IP fractions were analyzed by SDS-PAGE and co-purified proteins were stained by Coomassie blue (upper panel) or immunodetected using anti-His, anti-Strep and anti-FLAG antibodies (lower panels). Molecular weight markers (Mw, in kDa) are indicated on left. The pull-down assay has been performed in triplicate, and a representative experiment is shown.

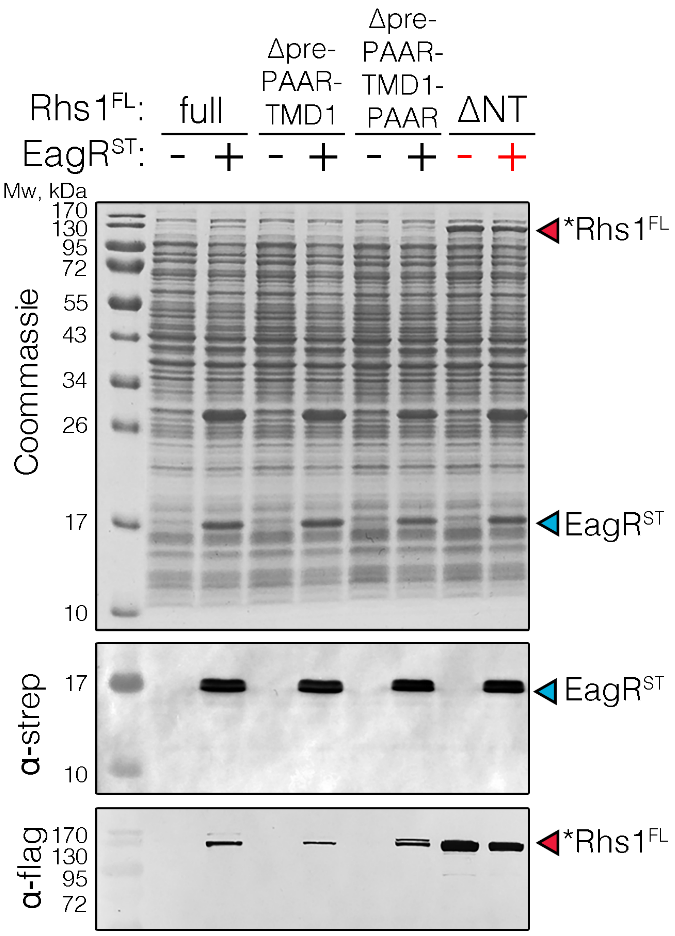

Supplementary Figure 4 | EagR-dependent stability of Rhs1 truncated variants. Total lysates of *E. coli* cells producing C-terminally FLAG-tagged Rhs1 full-length (full) or truncated variants as indicated, in absence (-) or presence (+) of Strep-tagged EagR ($EagR^{ST}$) were analyzed by SDS-PAGE and Coomasssie blue staining (upper panel) and anti-Strep and anti-FLAG immunodetection (middle and lower panels, respectively). Molecular weight markers (Mw, in kDa) are indicated on left. The positions of EagR (blue arrowheads) and of Rhs1 truncations (red arrowheads) are indicated on right. The experiment has been performed in triplicate, and a representative experiment is shown.

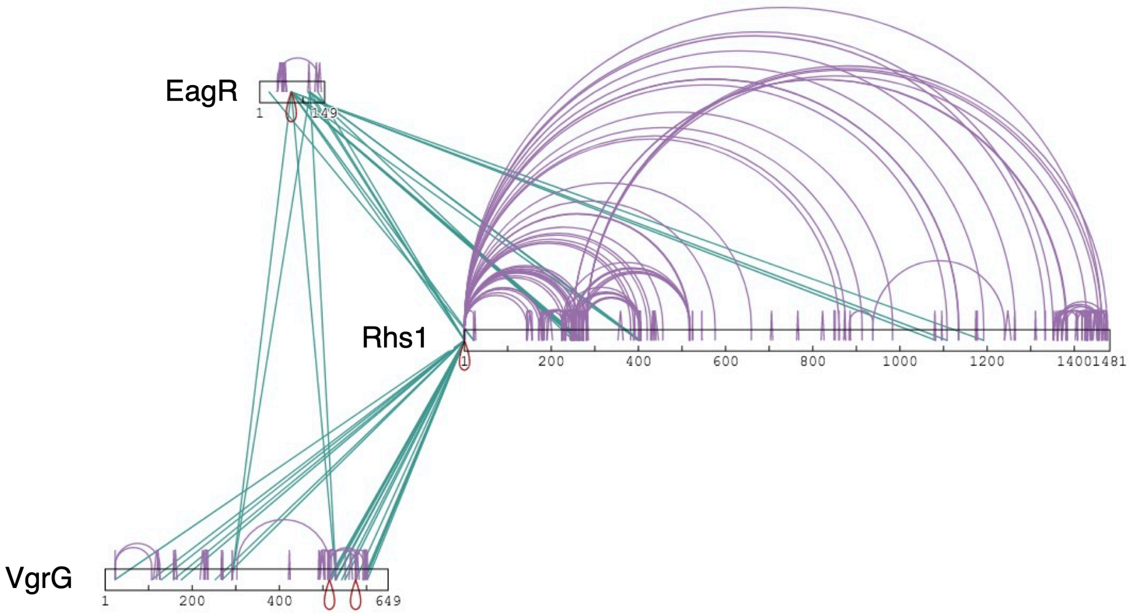

Supplementary Figure 5 | Schematic representation of inter-protein and intra-protein contacts of Rhs1, EagR and VgrG detected by cross-linking with NNP9 coupled to mass spectrometry analyses. The representation is made with the Crosslinkviewer software. Inter- and intra-protein contacts are shown in purple and green, respectively.

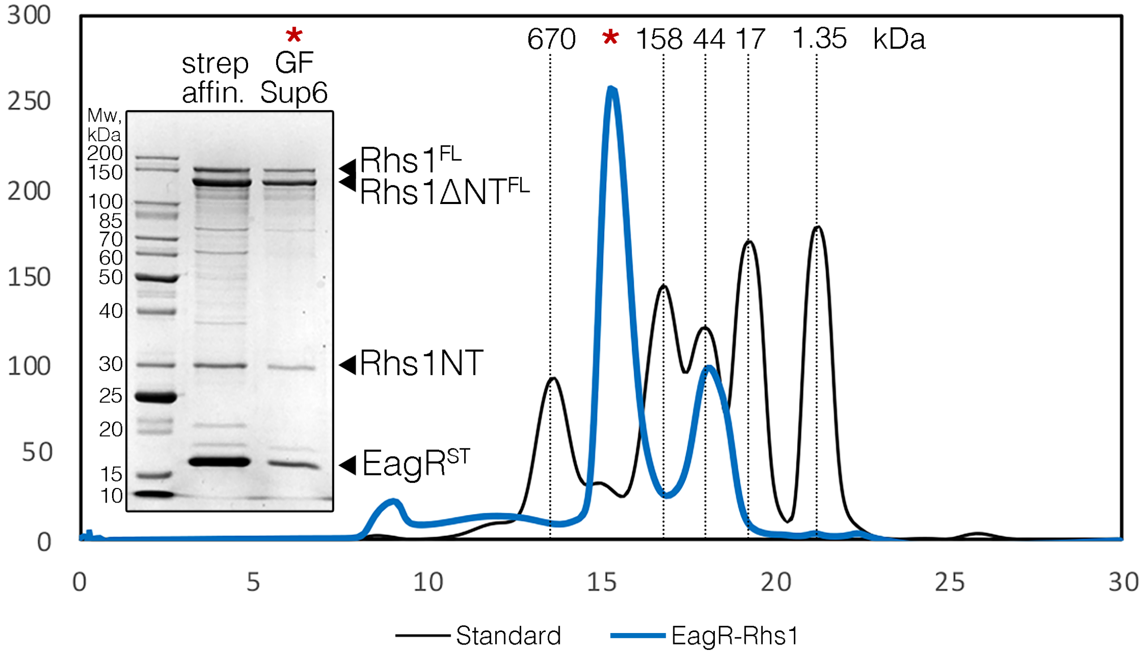

Supplementary Figure 6 | Purification of the Rhs1-EagR complex. Chromatogram of the purified *P. laumondii* Rhs1-EagR complex (blue line) on Superose6. The major peak (red star, *) corresponds to the purified complex whereas the 42-kDa peak corresponds to the EagR dimer. Molecular weight standards are shown as the black line. The inset on left shows the SDS-PAGE analysis and Coomassie blue staining of the fractions obtained after StrepTactin affinity (strep affin.) and gel filtration (GF, fraction corresponding to the * peak) chromatographies. Molecular weight markers (Mw, in kDa) are indicated on left.

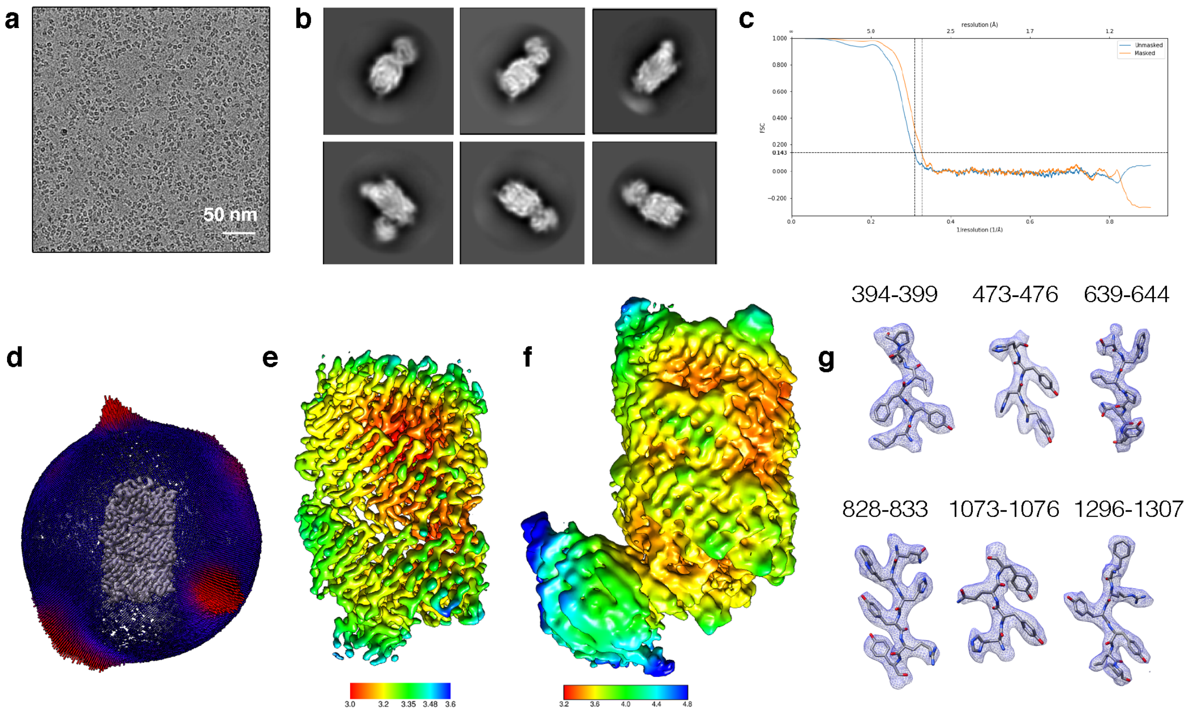

Supplementary Figure 7 | Structural modelling of the *P. laumondii* Rhs1 barrel by cryo-EM. (a) Cryo-electron micrograph of the Rhs1-EagR complex. The purified Rhs1-EagR complex has been deposited on eight independent grids, and a representative field is shown. (b) Representative 2D classes of the Rhs1-EagR complex after 2D classification in Relion 3.1. (c) Fourrier Shell Correlation (FSC) of the final Rhs1 3D reconstruction. The unmasked map crosses the 0.143 FSC threshold at 3.23 Å, while the masked map crosses at 3.05 Å. (d) Orientation distribution of particles used for 3D reconstruction in Relion 3.1. The number of particles in each orientation is proportional to the plotted bars on the Euler sphere around a representation of the Rhs1 cryo-EM map. (e) Final 3D cryo-EM masked map of Rhs1 coloured by local distribution. (f) Global mask of the Rhs1-EagR complex coloured by local resolution. (g) Representative regions of the Rhs1 cryo-EM map in mesh layout, with respective modelled residues shown as a stick representation.

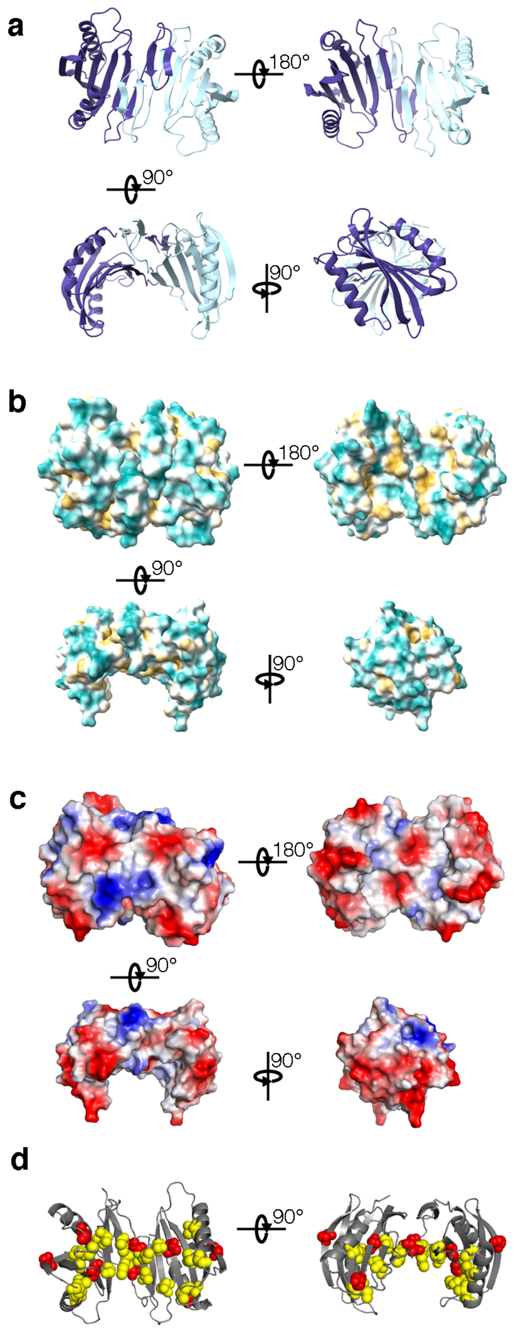

Supplementary Figure 8 | *P. laumondii* Eag structural model. The model was generated using the Galaxy Homomer server and the *P. aeruginosa* EagR protein (PDB: 1TU1) as template, imposing a dimeric oligomeric state. (a) Ribbon representation. The two monomers are colored in dark blue and cyan. (b) Surface hydrophobicity representation in the range of colors, from cyan (hydrophilic) to sand (hydrophobic) based on Kyte-Doolittle scale. (c) Electrostatic surface representation (blue: positive charges, red, negative charge; grey, neutral or hydrophobic). (d) Mapping of the EagR residues in contact with Rhs1 on the EagR structural model. Residues identified by our cross-link mass spectrometry approach are shown in red while residues in yellow represent conserved residues between Eag members[1].

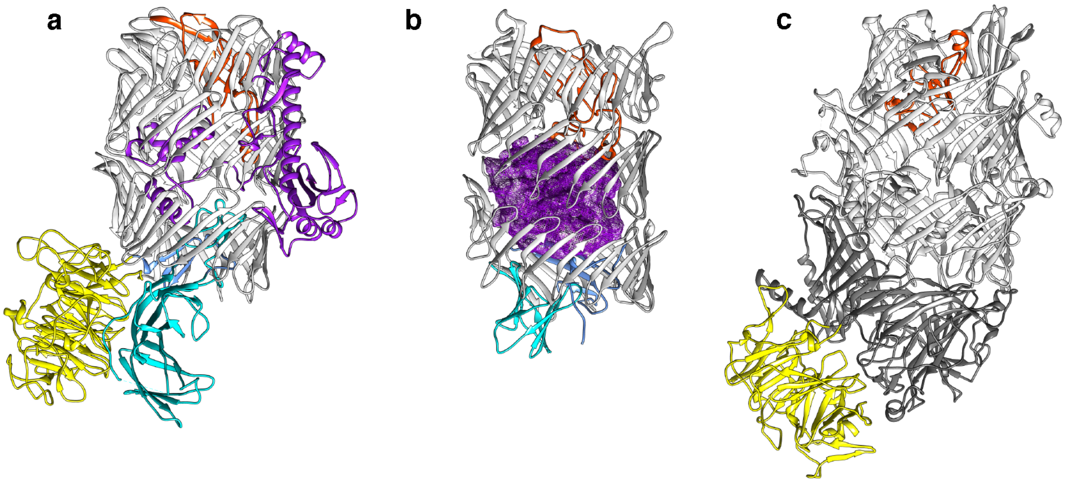

Supplementary Figure 9 | Structural comparison of teneurin (a), Rhs1 (b) and TcB-TcC complex (c). The N-terminal plug is shown in blue (Ig-like in cyan and seal in light blue), the β-barrel in grey (dark and light grey for TcB and TcC), the protease domain in red; the C-terminal extension (toxin for Rhs and Tc-toxins) in purple, and, when present, the β-propeller in yellow.

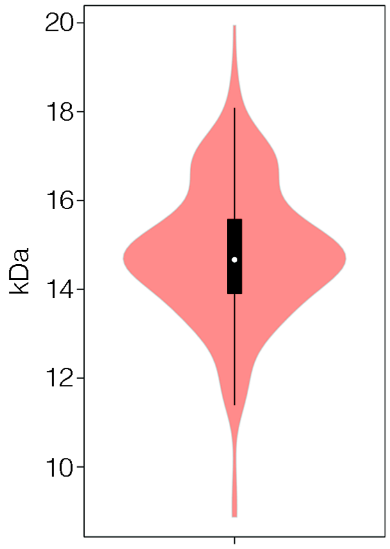

Supplementary Figure 10 | Violin plot distribution of the molecular weight (in kDa) of the Rhs^{CT} toxin extensions (*n*=222). Sequences from Rhs proteins were extracted, and the C-terminal extension domains were identified by the position of the conserved PxxxxDPxGL demarcation. The violin distribution (red surface) indicates the number of proteins (in the horizontal axis) with the indicated molecular weight (in kDa). The minima (8.868 kDa) and maxima (19.945 kDa) are shown. The central black bar indicates the first (13.915 kDa) and third (15.577) quartile, and the white dot indicates the median (14.666 kDa). The lower and upper whiskers indicate the 5^e (11.530 kDa) and 95^e (17.691 kDa) percentiles respectively.

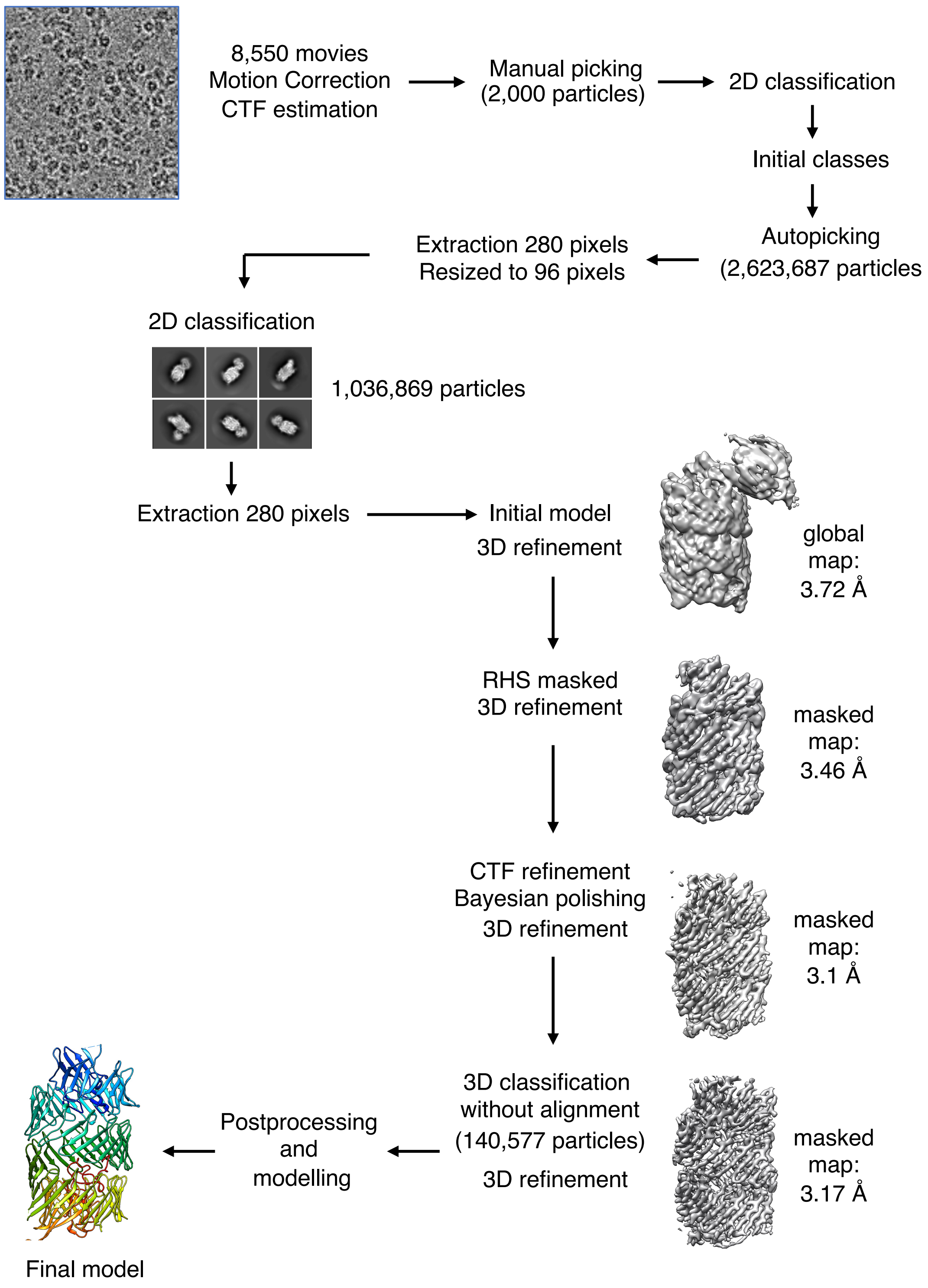

Supplementary Figure 11 | Flowchart of the cryo-EM processing procedure for the *P. laumondii* Rhs1-EagR complex, from data collection to the final model.

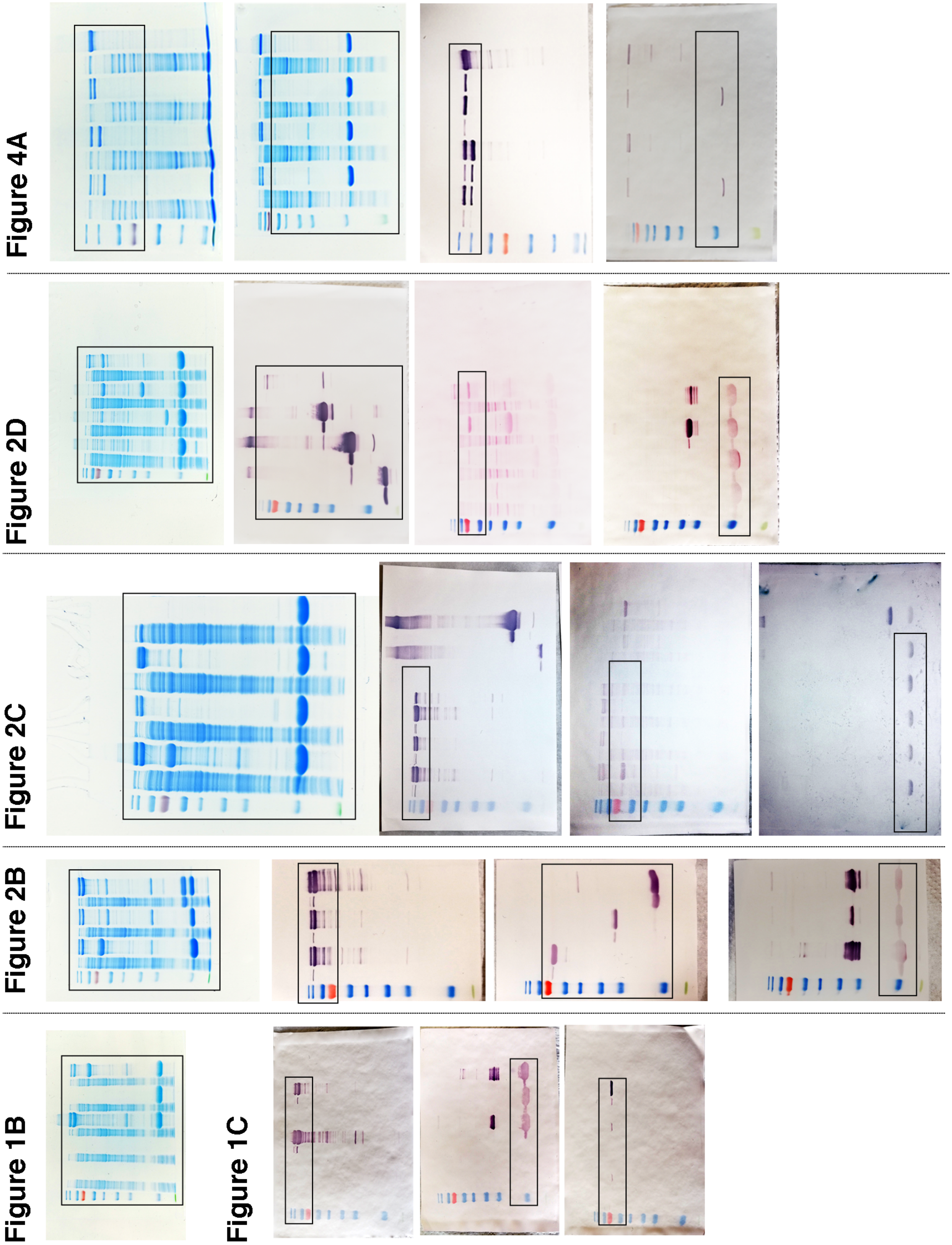

Supplementary Figure 12 | Uncropped gels and Western-blots shown in this study.

Figure S6

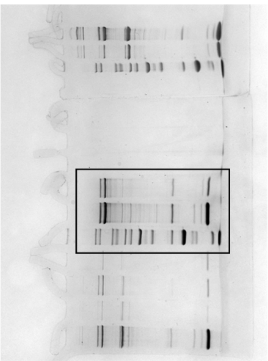

Figure S4

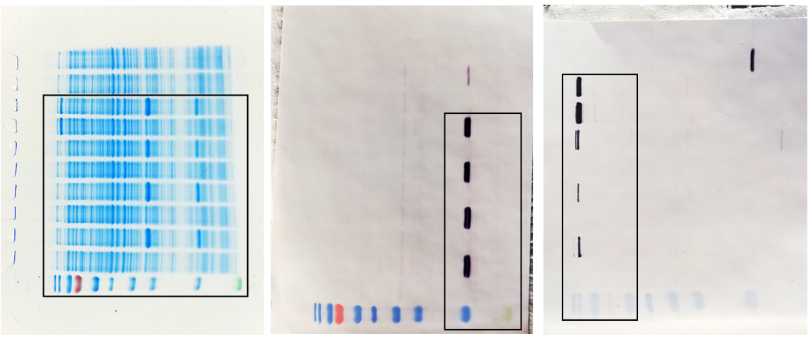

Figure S3

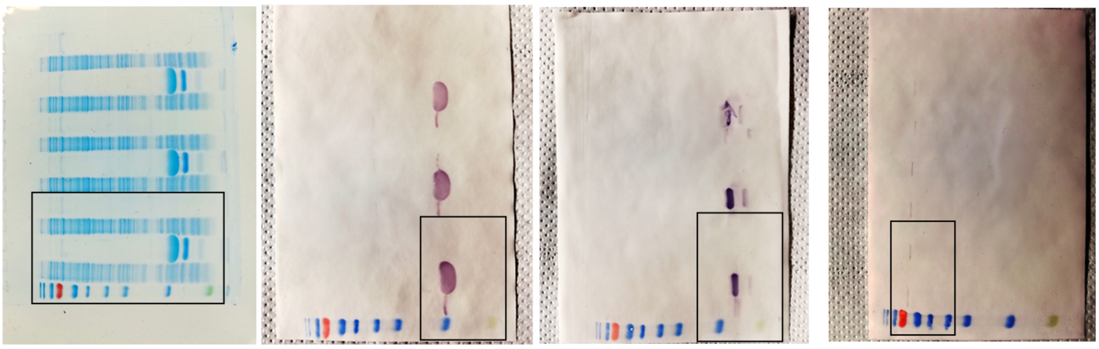

Supplementary Figure 12 (continued) | Uncropped gels and Western-blots shown in this study.

Supplementary Table 1. Inter-protein and intra-protein contacts of Rhs, EagR and VgrG detected by cross-linking with NNP9 coupled to mass spectrometry analyses.

Interacting proteins	Domains	Protein 1	Protein 2	Sum. score	Repetitions
**Inter-protein cross-links**					
EagR-Rhs1	EagR-prePAAR	EagR S74	Rhs S2	556.665845	3
		EagR T112	Rhs S2	146.7494	2
		EagR S117	Rhs S2	440.393313	3
		EagR S74	Rhs S23	248.418767	3
	EagR-TMD2	EagR S117	Rhs K252	191.604616	2
		EagR S74	Rhs K266	113.027074	2
EagR-VgrG	EagR-Gp5	EagR S74	VgrG S513	154.435659	2
		EagR S117	VgrG S513	344.787928	3
VgrG-Rhs1	Gp5-prePAAR	VgrG K495	Rhs S2	126.301191	2
		VgrG S513	Rhs S2	213.734864	3
		VgrG K558	Rhs S2	176.59158	2
		VgrG T575	Rhs S2	84.0028947	2
		VgrG K578	Rhs S2	223.263501	3
		VgrG S580	Rhs S2	142.234654	3
		VgrG K585	Rhs S2	271.78799	3
	Gp27-prePAAR	VgrG K6	Rhs S2	235.947861	2
		VgrG T112	Rhs S2	85.563533	2
		VgrG T252	Rhs S2	85.2255021	2
**Intra-protein crosslinks**					
VgrG	Gp27-Gp27	VgrG K6	VgrG K90	217.276464	3
	Gp5-Gp5	VgrG K558	VgrG K498	87.6341921	2
Rhs1	prePAAR-PAAR	Rhs S2	Rhs S145	133.284346	2
		Rhs S2	Rhs K173	258.140749	3
		Rhs S2	Rhs K181	213.484328	3
	prePAAR-TMD2	Rhs S2	Rhs K226	207.428304	3
		Rhs S2	Rhs K234	221.499631	3
		Rhs S2	Rhs K247	160.15346	3
		Rhs S2	Rhs K251	327.546684	3
		Rhs S2	Rhs K252	145.863835	2
		Rhs S2	Rhs K254	219.840478	3
		Rhs S2	Rhs S258	70.3621506	2
		Rhs S2	Rhs K266	203.446308	3
		Rhs S2	Rhs K275	165.808631	3

	prePAAR-barrel	Rhs S2	Rhs Y383	57.1368549	2
		Rhs S2	Rhs S390	75.1920292	2
		Rhs S2	Rhs Y421	63.8548761	2
		Rhs S2	Rhs Y429	76.2194561	2
		Rhs S2	Rhs T457	82.2626782	2
		Rhs S2	Rhs K519	187.744301	3
	PAAR-TMD2	Rhs K173	Rhs K251	79.7362991	2
	TMD2-barrel	Rhs K226	Rhs K519	141.095336	3
		Rhs K251	Rhs K519	73.8963874	2
		Rhs K254	Rhs K518	138.865154	3
		Rhs K266	Rhs K519	88.6816159	3
		Rhs T271	Rhs K519	78.3821213	2
	barrel	Rhs S885	Rhs S939	130.020977	2
	barrel-CT	Rhs K282	Rhs K1463	99.9313447	2
		Rhs K282	Rhs K1476	98.5609677	3
	CT	Rhs K1362	Rhs K1464	212.879594	2
		Rhs K1373	Rhs K1464	207.342656	2

Supplementary Table 2. Cryo-EM data collection, processing and model refinement statistics of the *P. laumondii* EagR-Rhs1 complex.

Data collection	EMD-13587, PDB 7PQ5
Microscope	Talos Arctica
camera	Gatan K2 Summit
Voltage (kV)	200
Magnification	45 000
Electron exposure (e− per Å^2)	1.32
Total Dose	49.75
Pixel size (Å)	0.93
Decofus range (um)	-0.5 to -2
Processing	
Symmetry imposed	C1
Micrographs number	8550
Initial particle images (no.)	1 036 869
Final particle images (no.)	140 577
Map resolution (Å)–(0.143 FSC threshold model)	3.1
Refinement and validation	
Map sharpening (B-factor) (Å−2)	75.76
Model composition	
No. of chains	1
Atoms (no.)	8527
Residues (no.)	1049
Ligands (no.)	0
Bond lengths (Å)	0.005
Bond angles (°)	1.178
Ramachandran favored %	95.28
Ramachandran allowed %	4.52
Ramachandran outliers %	0.19
Rotamers outliers %	2.92
MolProbity score	1.8
Clashscore	3.37
CC (mask)	0.87
CC (box)	0.85
CC (peaks)	0.82
CC (volume)	0.86

Supplementary Table 3. Strains, plasmids and oligonucleotides used in this study.

**Strains**	**Description and genotype**	**Source/References**
Escherichia coli K12		
DH5α	F-, Δ(*argF*-lac)U169 *phoA supE44 lacZ*ΔM15 *recA relA endA thi hsdR gyr*	Laboratory collection
BL21(DE3)	*dcm ompT hsdS gal λDE3*	Laboratory collection
Photorabdus laumondii		
TT01	WT *Photorhabdus laumondii*	2

**Plasmid**	**Description and main characteristics**	**Source/References**
pET-Duet1	pBR322 ColE1 *ori*, T7 promoter, Amp^R	Novagen
pET-His-VgrG	*P. laumondii vgrG* gene (*plu0355*) cloned into pET-Duet1, N-terminal 6×His tag, TEV cleavage	3
pET-His-VgrG-Δ27	*P. laumondii vgrG* gene deleted of the gp27-coding sequence cloned into pET-Duet1	This study
pET-His-VgrG-Δ27-OB	*P. laumondii vgrG* gene deleted of the gp27 and OB-fold-coding sequence cloned into pET-Duet1	This study
pRSF-Duet1	RSF1030 (NTP1) *ori*, T7 promoter, Kan^R	Novagen
pRSF-EagR-ST	*P. laumondii eagR* gene (*plu0354*) cloned into pRSF-Duet1, C-terminal Strep-tag	3
pCDF-Duet1	CloDF13 *ori*, T7 promoter, $Strep^R$	Novagen
pCDF-Rhs1*-FL	*P. laumondii rhs1* gene (*plu0353)* cloned into pCDF-Duet1, C-terminal FLAG tag, Tyr1351-to-Ala and Tyr1375-to-Ala substitutions	3
pCDF-Rhs1*-FL-D288N	Asp288-to-Asn substitutions in pCDF-Rhs1*-FL	This study
pCDF-Rhs1*-FL-D1338N	Asp1338-to-Asn substitution in pCDF-Rhs1*-FL	3
pCDF-Rhs1*-FL-D288N-D1338N	Asp288-to-Asn and Asp1338-to-Asn substitutions in pCDF-Rhs1*-FL	This study
pCDF-Rhs1-FL-ΔprePAAR-TMD1	*P. laumondii rhs1* gene deleted of the prePAAR-TMD1-coding sequence cloned into pCDF-Duet1, C-terminal FLAG tag, Tyr1351-to-Ala, Tyr1375-to-Ala, Asp1338-to-Asn substitutions	This study
pCDF-Rhs1-FL-ΔprePAAR-TMD1-PAAR	*P. laumondii rhs1* gene deleted of the prePAAR-TMD1-PAAR-coding sequence cloned into pCDF-Duet1, C-terminal FLAG tag, Tyr1351-to-Ala, Tyr1375-to-Ala, Asp1338-to-Asn substitutions	This study
pCDF-Rhs1-FL-ΔprePAAR-TMD1-PAAR-postPAAR	*P. laumondii rhs1* gene deleted of the prePAAR-TMD1-PAAR-postPAAR-coding sequence cloned into pCDF-Duet1, C-terminal FLAG tag, Tyr1351-to-Ala, Tyr1375-to-Ala, Asp1338-to-Asn substitutions	This study

pCDF-prePAAR-TMD1-PAAR-postPAAR-FL	*P. laumondii rhs1* prePAAR-TMD1-PAAR-postPAAR-coding sequence cloned into pCDF-Duet1, C-terminal FLAG tag	This study
pCDF-prePAAR-TMD1-PAAR-FL	*P. laumondii rhs1* prePAAR-TMD1-PAAR-coding sequence cloned into pCDF-Duet1, C-terminal FLAG tag	This study
pCDF-prePAAR-TMD1-FL	*P. laumondii rhs1* prePAAR-TMD1-coding sequence cloned into pCDF-Duet1, C-terminal FLAG tag	This study
pCDF-postPAAR-FL	*P. laumondii rhs1* postPAAR-coding sequence cloned into pCDF-Duet1, C-terminal FLAG tag	This study
pBAD33	Cloning vector, pACYC P15A *ori*, *araBAD* promoter, Cat^R	4
pBAD33-rbs	pBAD33 with ribosome-binding site consensus sequence inserted into MCS	This study
pBAD-Rhs1	*P. laumondii rhs1* gene (*plu0353*) cloned into pBAD33-rbs	This study
pBAD-Rhs1-D288N	Asp288-to-Asn substitutions in pBAD-Rhs1	This study
pBAD-Rhs1-D1338N	Asp1338-to-Asn substitution in pBAD-Rhs1	This study
pBAD-Rhs1-D288N-D1338N	Asp288-to-Asn and Asp1338-to-Asn substitutions in pBAD-Rhs1	This study

**Oligonucleotide**	**Sequence (5' to 3')**	**Purpose**
Plasmid construction [a]		
F-plVgrG3-386-Bmt	GGGC**GCTAGC**GCGATGGAGGGCCCGCAGAT	in pair with R-plVgrG3-Hind to clone C terminal fragments of VgrG
F-plVgrG3-485-Bmt	GGGCGCTAGCGGCAGCAAAACCCAGATGA	in pair with R-plVgrG3-Hind to clone C terminal fragments of VgrG
R-plVgrG-hind	GACTAAGCTTTCAGTTCACATTGACCTGTTTG	to clone VgrG or C terminal fragments of VgrG
F-plRhsPAAR-sac	GGAGGAGCTCATGGTGGACAGCATCGGCCC	in pair with R-plRhs-Sal to clone N terminally truncated Rhs1
F-plRhsTM2-sac	GGAGGAGCTCATGTCCGGTCAGGGAACTTACCTGG	in pair with R-plRhs-Sal to clone N terminally truncated Rhs1
F-plRhs-barrel-Sac	GGAGGAGCTCATGACGAAAAAATTCTTTACCGG	in pair with R-plRhs-Sal to clone N terminally truncated Rhs1
R-plRhs-Sal	GATCGTCGACTTTTCCTCCGCACTTTCTGATT	to clone Rhs or N terminally truncated Rhs1
F-plRhs-Sac	GGAGGAGCTCATGTCACTTGGTGATGAAATCG	to clone Rhs or N terminal fragments of Rhs1
R-plRhsTMD1-Sal	GATCGTCGACGTTCGTGACGGCCGAACTGG	in pair with F-plRhs-Sac to clone N terminal fragments of Rhs1
R-plRhsPAAR-Sal	GATCGTCGACGCCGATAAACACCGTTTTGG	in pair with F-plRhs-Sac to clone N terminal fragments of Rhs1
R-plTMPAARTM-Sal	GATCGTCGACCGTCGCTTCCCGGTAACGTTTT	in pair with F-plRhs-Sac to clone N terminal fragments of Rhs1
F-pBAD-MCS2	GATCCTCTAGAGTCGACCTGCAG	in pair with R-pBADsd-Sac to insert rbs sequence into pBAD33
R-pBADsd-Sac	GTACCGAGCTCCCTCCTGAATTCGCTAGCCCAAAAAA	in pair with F-pBAD-MCS2 to insert rbs sequence into pBAD33

Site directed mutagenesis [b]		
F-plRhs-D288N	AACCCGATTGACGTCACCACC	introduce Rhs1 D288N mutation
R-plRhs-D288	GCCGGTAAAGAATTTTTCGTCGC	introduce Rhs1 D288N mutation
F-plRhs-D1338N	AACCCTCTAGGGCTTGCTGGT	introduce Rhs1 D1338N mutation
R-plRhs-D1338	AATAAATTTCGTGGGATTTTGCACATAGC	introduce Rhs1 D1338N mutation

[a] Restriction site in bold.
[b] introduced mutation underlined.

Supplementary References

1. Ahmad, S., Tsang, K.K., Sachar, K., Quentin, D., Tashin, T.M., Bullen, N.P., Raunser, S., McArthur, A.G., Prehna, G., & Whitney, J.C. Structural basis for effector transmembrane domain recognition by type VI secretion system chaperones. *Elife* 9, e62816 (2020)
2. Duchaud, E., Rusniok, C., Frangeul, L., Buchrieser, C., Givaudan, A., Taourit, S., Bocs, S., Boursaux-Eude, C., Chandler, M., Charles, J.-F., *et al*. The genome sequence of the entomopathogenic bacterium *Photorhabdus luminescens*. *Nat Biotechnol* **21,** 1307–1313 (2003).
3. Jurenas, D., Payelleville, A., Roghanian, M., Turnbull, K.L., Givaudan, A., Brillard, J., Hauryliuk, V., & Cascales, E. *Photorhabdus* antibacterial Rhs polymorphic toxin inhibits translation through ADP-ribosylation of 23S ribosomal RNA. *Nucleic Acids Research* **49,** 8384–8395 (2021).
4. Guzman, L.M., Belin, D., Carson, M.J., & Beckwith, J. Tight regulation, modulation, and high-level expression by vectors containing the arabinose PBAD promoter. J Bacteriol 177, 4121–4130 (1995).
