# Supplementary material for: Mounting, structure and autocleavage of a type VI secretion-associated Rhs polymorphic toxin
Source: Nat Commun. 2021 Dec 1;12:6998. doi: 10.1038/s41467-021-27388-0 (PMC8636562; doi:10.1038/s41467-021-27388-0)
Supplement: Supplementary file 2 — Reporting summary [file 41467_2021_27388_MOESM2_ESM.pdf]

nature portfolio

Corresponding author(s): Dukas Jurenas
Rémi Fronzes
Eric Cascales

Last updated by author(s): Nov 8, 2021

Reporting Summary

Nature Portfolio wishes to improve the reproducibility of the work that we publish. This form provides structure for consistency and transparency in reporting. For further information on Nature Portfolio policies, see our Editorial Policies and the Editorial Policy Checklist.

Statistics

For all statistical analyses, confirm that the following items are present in the figure legend, table legend, main text, or Methods section.

n/a	Confirmed	
☐	☒	The exact sample size (*n*) for each experimental group/condition, given as a discrete number and unit of measurement
☒	☐	A statement on whether measurements were taken from distinct samples or whether the same sample was measured repeatedly
☒	☐	The statistical test(s) used AND whether they are one- or two-sided<br>*Only common tests should be described solely by name; describe more complex techniques in the Methods section.*
☒	☐	A description of all covariates tested
☒	☐	A description of any assumptions or corrections, such as tests of normality and adjustment for multiple comparisons
☒	☐	A full description of the statistical parameters including central tendency (e.g. means) or other basic estimates (e.g. regression coefficient) AND variation (e.g. standard deviation) or associated estimates of uncertainty (e.g. confidence intervals)
☒	☐	For null hypothesis testing, the test statistic (e.g. *F*, *t*, *r*) with confidence intervals, effect sizes, degrees of freedom and *P* value noted<br>*Give P values as exact values whenever suitable.*
☒	☐	For Bayesian analysis, information on the choice of priors and Markov chain Monte Carlo settings
☒	☐	For hierarchical and complex designs, identification of the appropriate level for tests and full reporting of outcomes
☒	☐	Estimates of effect sizes (e.g. Cohen's *d*, Pearson's *r*), indicating how they were calculated

Our web collection on statistics for biologists contains articles on many of the points above.

Software and code

Policy information about availability of computer code

Data collection	SerialEM 3.8: cryo-EM data collection

Data analysis	Bioinformatics analyses:<br>- BlastP<br>- JalView 2.11.1.4<br>- TMHMM v2.0<br>- ProtScale (ExPasy)<br><br>Structural modeling:<br>- GalaxyWEB<br>- AlphaFold2<br><br>Cryo-EM data analysis:<br>- Relion 3.1<br>- Chimera (Tcl 8.6.10 & TK 8.6.10)<br>- ChimeraX 0.93<br>- ISOLDE (built in ChimeraX)<br>- Coot 0.9.6<br>- Phenix 1.17.1<br><br>Proteomics data analysis:<br>- MassSpec Studio v2.4 (and E-Value Generator algorithm)

For manuscripts utilizing custom algorithms or software that are central to the research but not yet described in published literature, software must be made available to editors and reviewers. We strongly encourage code deposition in a community repository (e.g. GitHub). See the Nature Portfolio guidelines for submitting code & software for further information.

Data

Policy information about availability of data

All manuscripts must include a data availability statement. This statement should provide the following information, where applicable:

- Accession codes, unique identifiers, or web links for publicly available datasets
- A description of any restrictions on data availability
- For clinical datasets or third party data, please ensure that the statement adheres to our policy

The final cryo-EM density map of the P. laumondii Rhs1-EagR complex has been deposited to the Electron Microscopy Data Bank (EMDB) under the accession code EMD-13587 (https://www.emdataresource.org/EMD-13587). The final atomic model was deposited into the Protein Data Bank (PDB) under the accession code 7PQ5 (http://doi.org/10.2210/pdb5XRN/pdb). The proteomics data have been deposited to the ProteomeXchange Consortium via the PRIDE partner repository with the dataset identifier PXD028652 (http://dx.doi.org/10.6019/PXD025264). The data are provided in the Supplementary Information files or can be obtained from the corresponding authors upon request. Uncropped gels and Western-blots are shown in Supplementary Fig. 12.

Field-specific reporting

Please select the one below that is the best fit for your research. If you are not sure, read the appropriate sections before making your selection.

☒ Life sciences ☐ Behavioural & social sciences ☐ Ecological, evolutionary & environmental sciences

For a reference copy of the document with all sections, see nature.com/documents/nr-reporting-summary-flat.pdf

Life sciences study design

All studies must disclose on these points even when the disclosure is negative.

Sample size	No statistical method was used to determine sample sizes. However, sample sizes indicated in figure legend were such that standard error of the mean were within a confidence interval of 99 %.
Data exclusions	No data were excluded from the analysis.
Replication	Experiments were done, at least, in triplicate, each from three independent biological samples, with identical results. For Coomassie-stained gel, immunoblots, and Toxicity assays, a representative result is shown.
Randomization	All the experiments were performed with a random selection of clones. All experiments were performed with clonal poplations, issued from single colonies. All samples were randomly allocated into experimental groups.
Blinding	No blinding was performed, as acquisition and analysis methods required human intervention.

Reporting for specific materials, systems and methods

We require information from authors about some types of materials, experimental systems and methods used in many studies. Here, indicate whether each material, system or method listed is relevant to your study. If you are not sure if a list item applies to your research, read the appropriate section before selecting a response.

Materials & experimental systems

n/a	Involved in the study
☐	☒ Antibodies
☒	☐ Eukaryotic cell lines
☒	☐ Palaeontology and archaeology
☒	☐ Animals and other organisms
☒	☐ Human research participants
☒	☐ Clinical data
☒	☐ Dual use research of concern

Methods

n/a	Involved in the study
☒	☐ ChIP-seq
☒	☐ Flow cytometry
☒	☐ MRI-based neuroimaging

Antibodies

Antibodies used

- monoclonal anti-His antibody: Proteintech clone 1B7G5; catalogue number 66005-1-Ig, dilution 1/5,000
- Strep-Tag Classic: Bio-Rad clone Strep-tag II; catalogue number MCA2489, dilution 1/1,000
- anti-FLAG antibody: Sigma-Aldrich clone M2; catalogue number F3165, dilution 1/1,000
- secondary goat antibodies coupled to Alkaline Phosphatase: AffiniPure, Jackson ImmunoResearch; catalogue number 115-055-003, dilution 1/5,000

Validation

All antibodies were validated by Western-blot, with samples producing or not, the tagged proteins. Exemples of validation from cells that do not produce the tagged protein is shown in Figure 1c.
